# Acute cardiovascular health effects in a panel study of personal exposure to traffic-related air pollutants and noise in Toronto, Canada

**DOI:** 10.1038/s41598-020-73412-6

**Published:** 2020-10-07

**Authors:** Rita Biel, Coraline Danieli, Maryam Shekarrizfard, Laura Minet, Michal Abrahamowicz, Jill Baumgartner, Rick Liu, Marianne Hatzopoulou, Scott Weichenthal

**Affiliations:** 1 Department of Epidemiology, Biostatistics and Occupational Health, Faculty of Medicine, McGill University, 1020 Pine Ave West, Montreal, QC H3A 1A2 Canada; 2 Centre for Outcomes Research and Evaluation, Research Institute of the McGill University Health Centre, McGill University, 5252 de Maisonneuve Blvd. West, Montreal, QC H4A 3S5 Canada; 3 Department of Civil Engineering, University of Toronto, Galbraith Building Room 305F, 35 George St, Toronto, ON M5S 1A4 Canada; 4 Institute for Health and Social Policy, McGill University, Charles Meredith House, 1130 Pine Ave West, Montreal, QC H3A 1A3 Canada

**Keywords:** Environmental sciences, Cardiology, Risk factors

## Abstract

Urban populations are often simultaneously exposed to air pollution and environmental noise, which are independently associated with cardiovascular disease. Few studies have examined acute physiologic responses to both air and noise pollution using personal exposure measures. We conducted a repeated measures panel study of air pollution and noise in 46 non-smoking adults in Toronto, Canada. Data were analyzed using linear mixed-effects models and weighted cumulative exposure modeling of recent exposure. We examined acute changes in cardiovascular health effects of personal (ultrafine particles, black carbon) and regional (PM_2.5_, NO_2_, O_3_, O_x_) measurements of air pollution and the role of personal noise exposure as a confounder of these associations. We observed adverse changes in subclinical cardiovascular outcomes in response to both air pollution and noise, including changes in endothelial function and heart rate variability (HRV). Our findings show that personal noise exposures can confound associations for air pollutants, particularly with HRV, and that impacts of air pollution and noise on HRV occur soon after exposure. Thus, both noise and air pollution have a measurable impact on cardiovascular physiology. Noise should be considered alongside air pollution in future studies to elucidate the combined impacts of these exposures in urban environments.

## Introduction

Cardiovascular diseases (CVD), including ischemic heart disease and stroke, are the leading causes of death globally^1^. Air pollution is a well-known risk factor for cardiovascular disease and related mortality, accounting for 27% of deaths from heart disease and 34% of deaths from stroke^2^. Furthermore, the detrimental role of noise as an environmental stressor and its impact on cardiovascular disease is gaining recognition. Epidemiologic studies have linked exposure to persistent environmental noise with subclinical cardiovascular outcomes including arterial hypertension, atrial fibrillation, arrhythmia and vascular dysfunction, in addition to cardiometabolic diseases such as atherosclerosis, ischemic heart disease, stroke and type II diabetes mellitus^3–5^. Long-term residential exposure to road traffic noise has also been associated with myocardial infarction in a dose-dependent manner^6^ as well as heart failure^7^, and cardiovascular disease mortality^8^, while short-term exposure to traffic noise may also trigger cardiovascular events^9^.

To date, little research has examined the importance of urban noise exposure as a co-occurring environmental stressor that may confound or modify the adverse health impacts of traffic-related air pollutants (TRAPs), particularly among panel studies that assess short-term exposure in relation to acute cardiovascular outcomes. Preclinical outcomes including short-term changes in endothelial function (RHI; reactive hyperemia index, a measure of vascular function), blood pressure and heart rate variability (HRV; a measure of autonomic heart function) are acknowledged as important physiological mechanisms through which air pollution and noise may trigger cardiovascular events^10–12^. Fine particulate air pollution (PM_2.5_) and gaseous pollutants have been studied most often^11,13^. Yet, other pollutants such as ultrafine particles (UFPs) and black carbon (BC) may also provide important information about the influence of traffic pollution on acute and subacute cardiovascular responses, as these pollutants have high spatial variability and are known to be elevated in high-traffic areas, particularly areas impacted by diesel vehicles^14^. In this study, our objective was to examine the acute cardiovascular health effects of personal exposures to UFPs, BC, and noise along with other regional air pollutants including PM_2.5_, O_3_, and NO_2_. Furthermore, our objective was to evaluate the extent to which relationships between TRAPs and subclinical cardiovascular outcomes may be confounded by urban environmental noise using personal exposure measurements in a repeated-measures panel study design.

## Results

### Participant characteristics

In total, 46 adults (33 women and 13 men; mean age = 24 years) were enrolled and contributed a total of 87 sets of measurements between May and August, 2016. Most participants completed two study days (N = 41 participants, 89%) and all 46 completed one visit during which HRV was measured. Individual visits were separated by a mean of 14.7 days (range: 5 – 42 days). Participant characteristics are presented in Table 1. Participants were generally not obese and only two subjects reported a history of cardiovascular problems, including high blood pressure and heart flutters. None reported exposure to environmental tobacco smoke in the last 24 h before measurements. On average, morning baseline blood pressures, taken in a resting position prior to exposure measurements, were reflective of a non-hypertensive population (mean SBP = 115.1 mmHg, sd = 13.8; mean DBP = 65.1 mmHg, sd = 8.0). Similarly, means and distributions of RHI and HRV parameters did not reflect any aberrations in morning baseline endothelial function or HRV that would be suggestive of chronic cardiovascular illness (Supplementary Table S1). One participant had missing HRV measures at baseline and following exposure measurements, but some measures on occasions in between were collected and retained in the repeated measures analysis. Three additional participants were missing data for RHI, and two additional participants did not report past 24-h alcohol intake on one of their visits.

**Table 1:** Characteristics of the study participants (n = 46).

Characteristic	Category	n	Mean (sd) or %
Age	All	46	24.2 (8.2)
Sex	Males	13	28.3
	Females	33	71.7
Body mass index (kg/m^2^)^^^	All	46	22.5 (3.8)
	< 25 kg/m^2^	40	87.0
	≥ 25 kg/m^2^	6	13.0
Racial Group	Asian	26	56.5
	Caucasian	10	21.7
	South Asian or Pakistani	4	8.7
	Latin, Latino or Latino American	3	6.5
	Mixed race	2	4.3
	Black	1	2.2
History of cardiovascular problems*	Yes	2	4.3
Regular medication use**	Yes	10	21.7
Past 24-h alcohol consumption (visit 1) ***	Yes	5	11
Past 24-h alcohol consumption (visit 2)^^^^	Yes	2	5
Past 24-h caffeine consumption (visit 1)	Yes	16	35
Past 24-h caffeine consumption (visit 2)^^^^	Yes	15	37
Time between visits (days) ^^^^	All who completed both visits	41	14.7 (6.8)

^^^Body mass index (BMI) cutoff was chosen according to Canadian risk threshold guidelines defining BMI ≥ 25 kg/m^2^ as overweight or obese^68^. *High blood pressure (1 participant), history of heart flutters (1 participant). **Birth control (7 participants), Wellbutrin, Cymbalta/Wellbutrin/Abilify (2 participants), Claritin for allergies (1 participant). ***Not reported by 2 participants. ^^^^5 participants did not do the second visit. There was no reported environmental smoke exposure (if anyone has smoked inside the home or in their vicinity in the past 24 h).

### Personal and regional exposures to air pollution and noise

During the visits, participants were monitored for a daily mean of 4.9 h (sd = 0.75, median: 5 h, range 2.5—7 h), and spent a mean of 4.6 h outdoors during each visit (median: 4.8 h, range: 1.4– 6.5 h). Personal and fixed-site pollutant concentrations varied considerably throughout our study period, and we observed the highest variation in exposure for personal UFPs and BC (Table 2). Correlations between air pollutants were low to moderate. The highest correlations were between fixed-site PM_2.5_ and O_x_ (r = 0.72 (daily average) and r = 0.50 (30-min average)) and PM_2.5_ and O_3_ (r = 0.65 (daily average) and r = 0.45 (30-min average). Weaker correlations were found between personal BC and noise (r = 0.39 for daily average and r = 0.30 for 30-min average), BC and PM_2.5_ (r = 0.34, daily average), between UFPs and noise (r = 0.27, daily average), and between PM_2.5_ and NO_2_ (r = 0.35, daily average) (Supplementary Table S2). In the repeated measures analysis of HRV, 9.1%, 3.0% and 22.1% of all values were missing for UFPs, BC and noise, respectively, mostly due to instrument failure either for the entire visit or for some measurements during the visit (percentages represent missingness among all occasions recorded during the 46 completed HRV visits). In the baseline to follow-up analysis, missingness for UFPs, BC, and noise was 6.9%, 8.0%, 5.7% of the 87 completed visits, respectively. All missing values were excluded. In the following summary of our results, we focus on the personally collected pollutants (i.e. UFPs, BC, noise) and invite the reader to refer to the supplementary material for a summary of fixed-site regional pollutant associations.

**Table 2:** Distribution of personal and regional fixed-site daily average and 30-min average exposures to air pollution, personal exposure to noise, and environmental variables.

Exposure measure	n	Mean (sd)	5%	25%	Median	75%	95%	IQR*
**Daily visit average (87 study visits)**								
Personal exposures								
UFPs (particles/cm^3^)	81	20,480 (18,338)	2,918	7,742	16,560	22,631	57,501	14,890
Black carbon (ng/m^3^)	84	1,872 (1,435)	537	947	1,233	2,411	4,560	1,464
Noise (dBA)	82	66.9 (3.9)	60.7	64.2	67.3	69.4	73.8	5.2
Fixed-site exposures								
PM_2.5_ (µg/m^3^)	87	7.8 (3.2)	3.0	5.2	7.8	10.7	12.8	5.5
NO_2_ (ppb)	87	11.8 (3.0)	7.5	10.0	11.3	12.7	17.4	2.7
O_3_ (ppb)	87	27.6 (7.0)	17.5	21.2	27.4	33.3	36.8	12.2
O_x_ (ppb) **	87	22.2 (4.7)	15.1	18.4	22.2	25.8	28.7	7.4
Environmental variables								
Temperature (˚C)	87	23.4 (3.3)	16.6	21.8	24.1	25.7	26.9	3.9
Relative humidity (%)	87	51.5 (10.4)	34.2	42.4	53.6	57.8	66.7	15.4
**30-min average (46 study visits)**								
Personal exposures								
UFPs (particles/cm^3^)	420	19,733 (32,402)	76.9	4,385	12,185	22,336	65,940	17,950
Black carbon (ng/m^3^)	448	1,821 (2,873)	187	661	1,176	1,897	5,025	1,236
Noise (dBA)	360	66.9 (6.5)	53.9	63.0	68.1	71.4	76.0	8.4
Fixed-site exposures								
PM_2.5_ (µg/m^3^)	462	7.7 (5.6)	2.1	3.5	5.6	11.4	16.8	7.9
NO_2_ (ppb)	462	10.5 (3.5)	5.1	8.2	10.5	12.3	15.7	4.0
O_3_ (ppb)	462	32.3 (11.6)	16.3	23.6	31.1	38.6	56.4	15.1
O_x_ (ppb) **	462	24.9 (7.3)	14.3	19.8	24.3	28.5	39.3	8.7
Environmental variables								
Temperature (˚C)	462	25.0 (3.8)	17.9	22.9	25.4	27.2	31.0	4.3
Relative humidity (%)	462	43.8 (13.9)	25.9	33.1	42.2	51.7	73.5	18.6

*IQR = interquartile range. **Oxidant capacity of NO_2_ and O_3_ estimated as O_x_ = (1.07*NO_2_ + 2.075*O_3_)/3.145.

### Associations between pollution and acute (baseline to follow-up) within-person changes in cardiovascular outcomes

**Figure 1:**
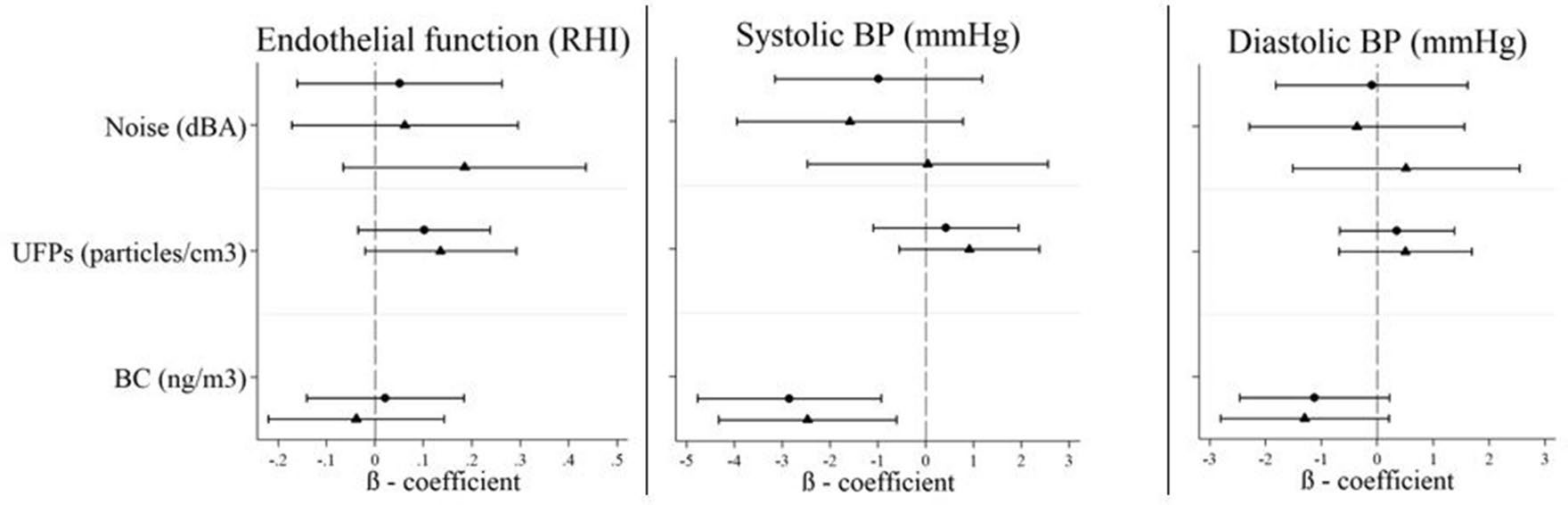
Associations between daily average pollutant measurement; and baseline to follow-up changes in endothelial function, systolic blood pressure and diastolic blood pressure, in single-pollutant (circle) and two-pollutant (triangle) models. *RHI* reactive hyperemia index. Multivariable models with random subject intercepts, adjusted for continuous temperature (degrees Celsius), alcohol intake (yes/no) and caffeine intake (yes/no) in the last 24 h. β-coefficients represent the change per IQR increase in exposure in a single-pollutant model (filled circle) or a two-pollutant (filed triangle) model (i.e. air pollutant + noise). β-coefficients for noise are shown in the following order: noise estimate in a single-pollutant model, followed by the noise estimate in two-pollutant models with UFPs and BC, respectively. Refer to Supplementary Table S3 (single-pollutant models) and S5 (two-pollutant models).

**Figure 2:**
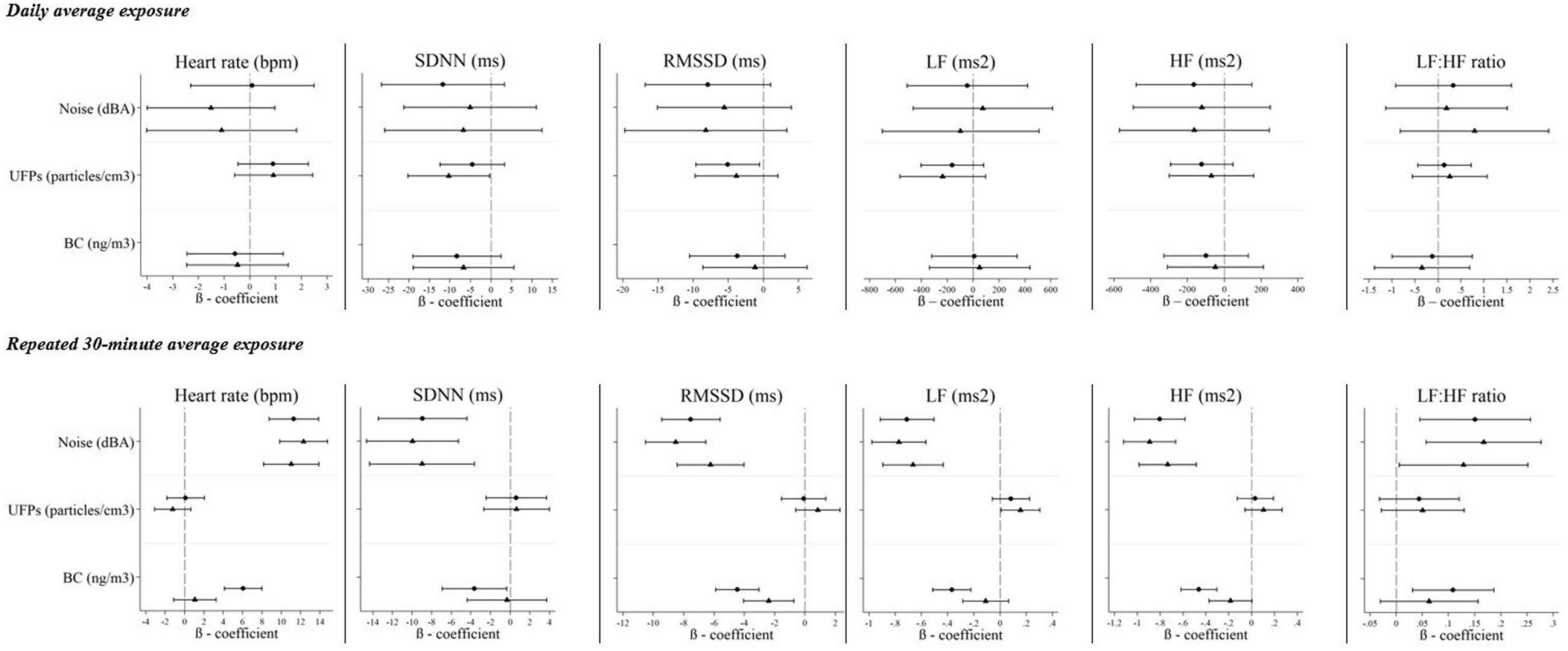
Associations between daily and 30-min average pollutant measurements and changes in heart rate and HRV parameters, in single pollutant (circle) and two-pollutant (triangle) models. *SDNN* standard deviation of normal-to-normal (NN) intervals, *RMSSD* root mean square of successive NN interval differences, *HF* high-frequency power, *LF* low-frequency power, *LF:HF* the LF to HF ratio, *bpm* beats per minute, *ms* milliseconds. Multivariate models with random subject intercepts in daily average models, and additional random slopes in 30-min average models, adjusted for continuous temperature (degrees Celsius), alcohol intake (yes/no) and caffeine intake (yes/no) in the last 24 h. In daily average models, β-coefficients represent the change per IQR increase in pollutant exposure in a single-pollutant model (circle) or a two-pollutant (triangle) model (i.e. air pollutant + noise). In 30-min average models. β-coefficients represent the change per IQR increase in pollutant exposure for heart rate, SDNN and RMSSD and the % change per IQR increase in exposure for LF, HF and LF:HF ratio, in a single-pollutant model (filed circle) or a two-pollutant (filed triangle) model (i.e. LF, HF and LF:HF outcomes were natural log transformed. UFPs were log transformed to base 5 and BC exposures were natural log transformed corresponding approximately to the IQRs for the untransformed exposure), β-coefficients for noise are shown in the following order: noise estimate in a single-pollutant model, followed by noise estimate in two-pollutant models with UFPs and BC, respectively. For daily average results, refer to Supplementary Tables S3, S4 (single-pollutant model) and S5, S6 (two-pollutant models). For 30-min average results, refer to Supplementary Table S7 (single-pollutant models) and S8 (two-pollutant models). Figures and tabulated results for fixed-site regional pollutants appear in the supplementary material.

Daily average personal noise exposure was not associated with changes from baseline, during the same day, in endothelial function (i.e. RHI), blood pressure and heart rate outcomes (Figs. 1 and 2). Similarly, daily average personal UFP and BC exposures were not associated with changes in endothelial function, but BC exposures were associated with reduced systolic and diastolic blood pressure. None of the air pollutants were associated with changes in heart rate on the same day for the daily average exposures. Consistent inverse associations were observed between personal UFP, BC, and noise exposures and the HRV parameters SDNN, RMSSD, and HF (Fig. 2). In general, the air pollution impacts on endothelial function and blood pressures were not confounded by noise as model coefficients for daily average air pollutants changed negligibly when noise was included as a covariate (Fig. 1). Conversely, evidence of confounding by noise was observed in models examining the association between air pollution and HRV (Fig. 2). In particular, adjustment for noise strengthened inverse associations between UFPs and SDNN (mean change of − 10.3 ms, 95% CI − 20.3 to + 0.35 per 14,890 particles/m^3^ with noise adjustment compared to − 4.55 ms, 95% CI − 12.4 to + 3.34 per 14,890 particles/m^3^ without adjustment) and LF (mean change of − 236.4 ms^2^, 95% CI − 568.5 to + 95.7 per 14,890 particles/m^3^ with noise adjustment compared to − 162.5 ms^2^, 95% CI − 404.7 to + 80.6 per 14,890 particles/m^3^ without noise adjustment), while the decrease in HF associated with UFPs was attenuated. Similarly, decreases in RMSSD associated with both UFP and BC exposures were also attenuated.

### Associations between pollution (30-min average exposure) and subacute cardiovascular outcomes

Higher short-term levels of personal noise exposures, over repeated 30-min exposure windows, were associated with marked decreases in concurrent HRV parameters SDNN, RMSSD, LF and HF (Fig. 2). In addition, higher 30-min noise exposures were also associated with increases in concurrent heart rate and the LF:HF ratio. Similarly, personal BC exposures were associated with decreases in concurrent SDNN, RMSSD, HF, and LF, while personal UFP exposures were not associated with any of the HRV parameters in single pollutant models. Noise estimates were largely unchanged in two-pollutant models compared to single pollutant models, similarly showing marked decreases across concurrent SDNN, RMSSD, HF and LF parameters, and marked increases with concurrent heart rate and the LF:HF ratio associated with noise. These results suggest that confounding by air pollutants does not explain adverse associations for these outcomes with noise.

With a few exceptions, associations identified between air pollutants and HRV parameters were generally attenuated when models were adjusted for personal noise exposures, approximately by a range of 13–91% (difference between the single-pollutant and two-pollutant coefficient as a percent of the single-pollutant coefficient value) (Supplementary Table S8). Confounding by noise was most apparent for associations between HRV parameters and BC exposures, resulting in attenuation of the single-pollutant estimates for BC exposures across all measures of HRV. However, the marked decreases in concurrent RMSSD, HF and LF in association with BC exposures observed in single-pollutant models were still present after noise-adjustment (% change of − 2.39 ms, 95% CI − 4.06 to − 0.73, % change of − 0.19 ms^2^, 95% CI − 0.37 to 0.002, and % change of − 0.11 ms^2^, 95% CI − 0.29 to 0.06, per 2.7-fold increase with noise adjustment, respectively).

### Sensitivity analyses

In additional sensitivity analyses, we examined if 30-min average air pollutant impacts on HRV were modified by sex, noise exposure (< 68.1 dBA vs ≥ 68.1 dBA) or O_x_ exposure (< 24.3 kg/m^2^ vs. ≥ 24.3 kg/m^2^). Overall, exposure to air pollutants had a greater impact on HRV (SDNN, RMSSD, HF) and heart rate among women (i.e. a stronger inverse association for HRV parameters and positive association for heart rate, with BC and PM_2.5_) (Supplementary Table S9). We also observed stronger inverse associations between noise and RMSSD among women (absolute change of − 8.9 ms, 95% CI − 11.3 to − 6.48 for women compared to − 5.25 ms, 95% CI − 8.37 to − 2.13 for men, per 8.4 dBA). Trends toward interactions between air pollutants and noise were also noted in models stratified by low vs. high noise exposure. Specifically, models stratified by noise level (i.e. equal to, or above the median 68.1 dBA) suggested that air pollution impacts on heart rate and HRV were, for some air pollutants (i.e. PM_2.5_, UFPs, O_3_ and O_x_), greater in subjects exposed to higher noise levels (Supplementary Table S9). Analyses across strata of O_x_ exposure (< 24.3 ppb vs ≥ 24.3 ppb) suggested that the relationship between PM_2.5_ and heart rate may be strongest when O_x_ is higher.

### Assessing potential cumulative exposure effects on HRV

**Figure 3:**
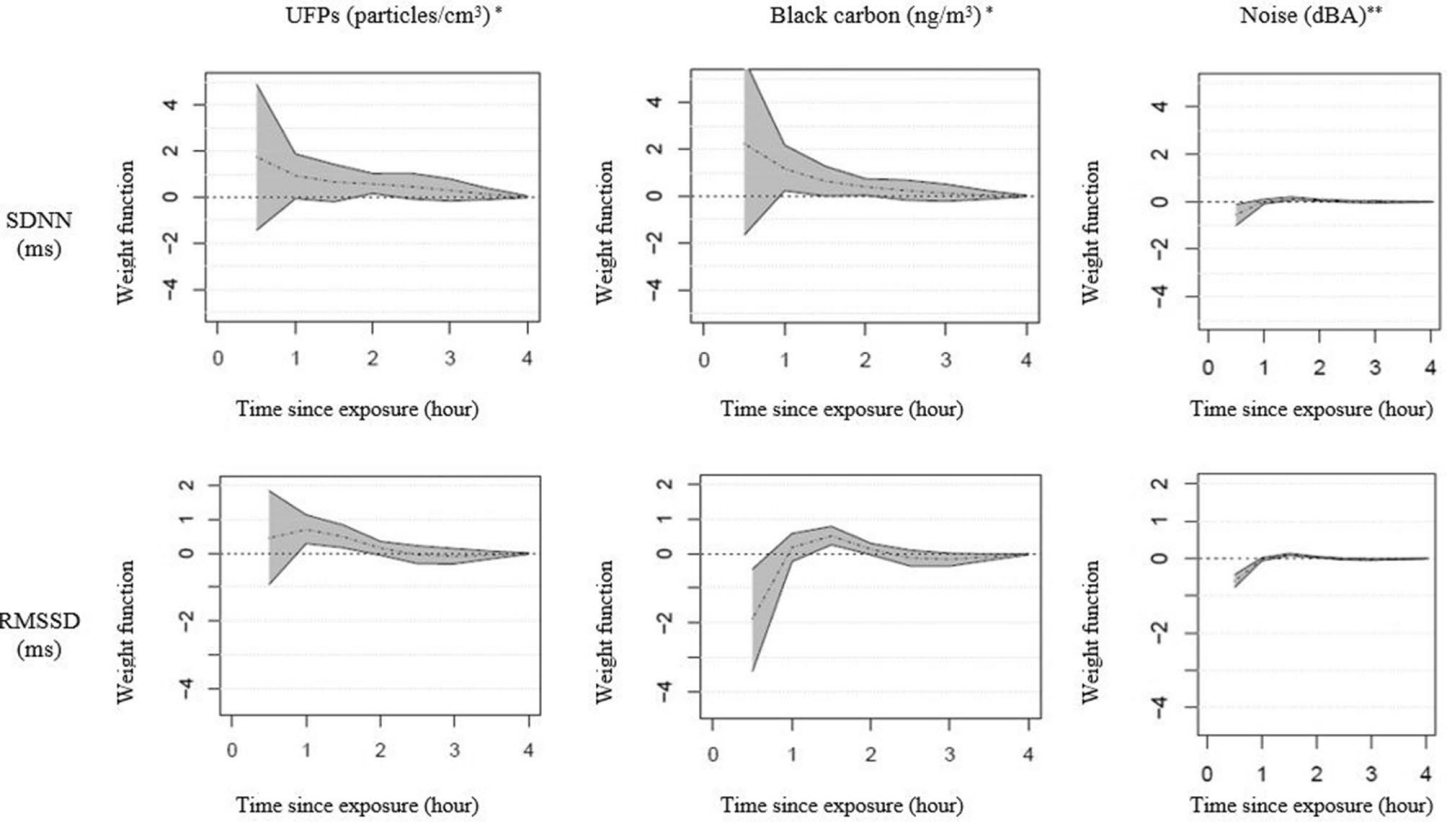
Timing of exposure, in hours since exposure occurs, having significant impact on SDNN and RMSSD parameters, estimated from a flexible weighted cumulative exposure mixed-effects model. Model results show the weight function (y-axis) and time since exposure (hours, x-axis). The weight function reflects exposure assigned in the past, therefore the higher the weight function for the exposure, the more importance the corresponding timing has on the outcome. *UFPs were log transformed to base 5 and BC exposures were natural log transformed, corresponding approximately to the IQRs for the untransformed exposure. **Weighted cumulative exposure mixed-effects model with noise as the only exposure. All models adjusted for continuous temperature (degrees Celsius), alcohol intake in the last 24 h (yes/no) and caffeine intake in the last 24 h (yes/no). Air pollutants were additionally adjusted for continuous noise exposure.

Figure 3 summarizes the results of weighted cumulative exposure to personal pollutants and shows how, for each association, the impact of past exposures changes with increasing time since exposure. Our results suggest that the impacts of recent short-term air pollution and noise levels on SDNN and RMSSD decrease quickly with time since exposure, but last for up to 2 h (Fig. 3). This pattern was most apparent for noise and BC with less of an impact observed for UFPs. Consistent with our mixed-effects modeling of most recent exposures, in weighted cumulative exposure models, we also found that (i) adjusting for noise attenuated the effects of personal air pollutants levels for HRV parameters, but (ii) noise-adjusted models nonetheless showed important associations.

## Discussion

This study examined the acute cardiovascular health effects of personal (UFPs, BC) and regional (PM_2.5_, NO_2_, O_3_, O_x_) measurements of air pollution exposures as well as the impact of personal noise exposures on these outcomes. We observed adverse changes in subclinical cardiovascular outcomes, including endothelial function and HRV, in response to increased air pollution and noise exposures. Importantly, our findings suggest that personal noise exposures can partly confound associations for air pollutants, primarily for HRV. Further, weighted cumulative exposure models suggested that the impacts of air pollution and noise on HRV occur very soon after exposure and then gradually decrease with increasing time since exposure, lasting for about two hours. This suggests that current HRV levels are affected by cumulative effects of exposure in the past two hours.

The physiological measures of cardiovascular disease measured in this study are widely hypothesized to be the mechanistic triggers linking air pollution and noise with adverse cardiovascular events^3,10–13,15^. In general, we noted several important findings. First, our results suggest that, where both noise and air pollution are independently associated with an acute cardiovascular outcome, neither pollutant completely explains the effect of the other. These findings corroborate results from several previous studies of concurrent exposure to traffic noise and air pollution on cardiovascular outcomes including prevalent hypertension and blood pressure ^16–18^, heart rate^17^, incident myocardial infarction^19^, hospital readmission for myocardial infarction^20^, all-cause mortality^20^, heart failure^7^, myocardial infarction mortality^21^ and CHD mortality^8^. These studies and others examining correlations between traffic noise and air pollution^22,23^ were the first to suggest that noise may confound traffic-related health effects in epidemiologic studies of air pollution, and we assessed this potential bias more precisely by using personal exposure measures. Our findings are consistent with previous studies and indicate that failing to adjust adequately for traffic noise may overestimate the impacts of air pollution on some acute and subacute physiological measures of cardiovascular disease.

Our findings are also consistent with other panel studies that show acute cardiovascular impacts of more commonly studied air pollutants (e.g. PM_2.5_ and other traffic pollutants)^10,11,24–36^, and panels that also measured UFP or BC exposures^24,25,27,30–32,37–43^, as well as noise^18,40,41,44–47^. To our knowledge, only four studies to date have examined both air pollution and noise exposures simultaneously with personal exposure measures^22,39–41^, three of which also measured acute outcomes^39–41^. Sarnat et al. (2014) conducted a study of 2-h highway commutes in Atlanta, USA among 42 adults and reported decreases in time-domain HRV parameters (SDNN, RMSSD) associated with in-vehicle PM_2.5_ mass, but noise was not independently associated with any of the measured outcomes^39^. Morishita et al. (2019) measured PM_2.5_, BC, total particle count (PC), and noise in relation to brachial blood pressure, aortic hemodynamics, endothelial function and HRV in a repeated measures intervention study of N95 respirators, and found that BC and PC were associated with lower RHI, while noise tended to decrease the LF:HF ratio; however, the authors noted that the study was not designed to examine the independent effects of noise^40^. Moshammer et al. (2019) measured PM_2.5_, UFPs, and noise exposure in 24 healthy students walking in Vienna, Austria under four settings to examine acute cardiovascular responses; along a busy road, along a busy road wearing ear plugs, in a park, and in a park but exposed to recorded traffic noise (65 dB) on headphones^41^. Noise levels were associated with reduced systolic blood pressure and lower HRV (SDNN, RMSSD, VLF, LF, HF parameters). Effects on HRV were strongest after 15 min of exposure and generally attenuated during the course of the walk. Air pollution effects were reported as somewhat inconsistent, and this study did not assess two-pollutant associations. Lastly, a study by Chang et al. (2015) conducted a panel in 66 young adults to investigate independent associations between personal noise exposure and air pollutants on changes in 24-h blood pressure and collected fixed-site measures of PM_2.5_ and NO_x_. They found that exposure to noise and air pollutants independently increased ambulatory blood pressure^18^.

To our knowledge, this is the first study to apply weighted cumulative exposure effects models to examine the importance of exposure timing in the acute cardiovascular impacts of air pollution and noise. Our results suggest that these exposures have a very short-term impact on HRV which is consistent with studies that reported, using different analytical methods, very short-term impacts of air pollutants on HRV, generally within 2 h after the exposure^24,25,41,48^, though others have suggested that these effects may persist for several hours after exposure^31,49^. Persistence of effects on HRV suggests that cardiovascular events could be triggered by exposures for some time after the exposure occurs. Our analysis stratified by sex suggested that the impacts of noise and air pollution exposures on HRV may be more pronounced among women. These results, together with those showing more pronounced effects of BC and PM_2.5_ on elevations in heart rate among women, suggest that women may be more susceptible to the acute autonomic impacts of air pollution and noise on HRV. One previous study showed the highest elevated risk of myocardial infarction onset was within one hour after exposure to traffic, and among sub-groups, women, patients aged 65 years or older, or those with existing comorbidity had the largest increases between time spent in traffic and acute myocardial infarction onset one hour later^50^. Kraus et al. (2013) found that sex modified associations between noise and HRV, where women appeared to respond at lower noise levels as shown by associations that were stronger with each 5-dBA increase in concurrent noise < 65 dBA for HR, HF and the LF:HF ratio, but there was no difference between women and men at higher noise > 65dBa^44^. In contrast, Zijlema et al. (2016) found a larger positive association between noise and HR in males than females^17^. Future studies should consider sex as an important potential modifying variable. Similarly, our assessment of effect modification by O_x_ suggested that associations between PM_2.5_ and heart rate were amplified by higher O_x_ exposures. This result is consistent with growing evidence suggesting that O_x_ modifies the cardiovascular health impacts of PM_2.5_ exposures^51,52^ and that regulatory actions targeting O_x_ could result in benefits related both to reductions in O_x_ directly as well as indirectly through reduced health impacts of PM_2.5_.

The specific mechanisms explaining how noise and air pollution impact cardiovascular physiology are thought to involve both inflammation and oxidative stress^53,54^. For example, noise induces the release of stress hormones and inflammatory signaling molecules leading to oxidative stress and vascular dysfunction^53^. Likewise, existing evidence suggests that exposures to high levels of particles from traffic sources may increase reactive oxygen species^55,56^ leading to systemic inflammation that contributes to progression of atherosclerosis. Furthermore, endothelial dysfunction is an important process in the development of cardiovascular disease and can promote vasoconstriction and myocardial ischemia in the coronary artery^57^. The single-cell endothelial lining of the internal surface of blood vessels maintains vascular homeostasis by controlling release of factors that influence vasoconstriction, vasodilation, smooth muscle cell proliferation, platelet aggregation and leukocyte adhesion^58^. Local vascular control depends on a balance between dilators and constrictors in the heart. One of the most important vasodilators is endothelium-dependent nitric oxide (NO)^58^, and endothelial dysfunction occurs when there is reduced production and/or availability of NO. Exposures such as air pollution may injure the endothelium over time and hasten its dysfunction, as NO may be removed by oxidative free radicals caused by air pollutants from for example, diesel exhaust, causing impaired vasodilation^34,36,59^. HRV is regulated by the autonomic nervous system and numerous studies have examined air pollution impacts on changes in HRV^11,24,31,35,38–41,60,61^ owing to the known relationship between reduced HRV and higher risks of cardiovascular morbidity and mortality^62^. Far fewer studies have examined the impact of noise on HRV^40,41,44,45^. Our results suggest that this physiological mechanism may be particularly important for noise impacts on cardiovascular health owing to the consistent relationship observed between noise and all measures of HRV in our study.

While our study had important strengths including detailed personal exposure measurements for noise and air pollution along multiple exposure days, it is important to recognize several limitations. First, we did not have personal exposure measures for some pollutants including PM_2.5_, O_3_, and NO_2_, thus our ability to detect associations for these pollutants might have been reduced. However, it is important to note that we did observe significant relationships between these pollutants and several cardiovascular outcomes. A second limitation relates to our evaluation of endothelial function. Specifically, endothelial function and blood pressure can be affected by salt intake and we did not have a measure of dietary salt intake in our study. However, in order to confound our results dietary salt intake would have to be systematically correlated with the environmental exposures monitored in our study. Such correlations seem implausible, especially for repeated within-person exposure measures. With respect to road traffic in urban environments, both noise and ground-level air pollution (such as UFPs, BC) may refer largely to the same sources, while levels of regional pollutants (PM_2.5_, O_3_) are more influenced by long-range transport and may represent additional sources^5^. The potential for bias amplification with inclusion of co-exposures in models where the pollutants have a common source, such as traffic, is a recognized concern when attempting multi-pollutant modelling due to co-linearity of exposures^63^. However, correlations between pollutants in our study were generally modest.

In general, our findings suggest that both noise and air pollution have a measurable impact on cardiovascular physiology including changes in endothelial function and heart rate variability. In some cases, noise can partly confound air pollution impacts on cardiovascular health, but both appear to have an important impact on specific parameters. Noise exposure should be considered alongside air pollution in future studies to better understand the combined impacts of noise and air pollution in urban environments and to better identify characteristics of susceptible populations. Consideration of both noise and air pollution will help to inform policy considerations and mitigation efforts aimed at reducing the impacts of environmental pollutants on cardiovascular health and may also inform preventive recommendations as part of risk profiles for susceptible individuals.

## Methods

### Study design and population

We conducted a repeated measures panel study including 46 non-smoking men and women, 18–60 years of age, in Toronto, Canada between May and August 2016. Participants were recruited from Toronto area universities, colleges and public spaces using posters and online ads. Eligible adults lived in homes without a cigarette smoker, were able to carry a small backpack of air sampling devices for at least 7 h, and provided informed consent. Eligible volunteers who reported a history of cardiovascular morbidity, including a previous myocardial infarction, history of interventional surgery such as heart bypass, or those living with a pacemaker, were excluded.

Each participant was asked to complete 2 days of repeated exposure and outcome measurements, scheduled at least 5 days apart. On the first day of study participation, staff administered a questionnaire recording age, sex, ethnicity, current medication use, alcohol and/or caffeine consumption for the past 24 h, recent illness, and any recent exposure to environmental tobacco smoke. Participants’ weight and height were measured to calculate body mass index (BMI in kg/m^2^). Participants were fitted with a backpack containing a GPS monitor and air and noise monitoring devices, prepared and calibrated by trained research staff. All participants spent study days in downtown Toronto near the University of Toronto campus, and were asked to engage in normal daytime activities on each day, up to 7 h extending from 9am to 4 pm, but to spend at least 2 h outdoors. As such, exposure measures reflected a combination of indoor and outdoor exposures, typical of urban life. Ethics approval was obtained from the institutional review board of the Faculty of Medicine at the University of Toronto and the protocol for involving humans was in accordance with institutional guidelines.

### Exposure assessment for air pollution and noise

Continuous personal exposures to ultrafine particles (UFPs, particles/cm^3^) and black carbon (BC, ng/m^3^) were measured using a miniature diffusion size classifier (DiSCmini, University of Applied Sciences Northwestern Switzerland, Windisch, Switzerland) and MicroAeth Model AE51 aethalometers (Magee Scientific, Berkeley, CA, USA), respectively. Noise (dBA) was measured using a personal noise dosimeter (Dose meter type 4448, Bruel & Kjaer, Nӕrum, Denmark) mounted on the shoulder strap of the backpack. Continuous air temperature and relative humidity data were recorded at the personal level using HOBO data loggers (Onset, Cape Cod, MA, USA). All measurements were done at a 1-min resolution except for 1-s intervals for UFPs.

Hourly average ambient PM_2.5_ (µg/m^3^), NO_2_ (ppb) and O_3_ (ppb) concentrations were also collected from a fixed monitoring site located in downtown Toronto on a balcony of the Walberg Chemical Engineering Building on College Street. We estimated redox-weighted oxidant capacity of NO_2_ and O_3_ from ambient measures of these pollutants by the weighted average of redox potentials: O_x_ = (1.07*NO_2_ + 2.075*O_3_)/3.145^52,64,65^.

### Measurement of cardiovascular outcomes

Baseline physiologic measures of endothelial function and blood pressure were taken in a resting, seated position in a quiet dimly lit room on the morning of the exposure period on each study day. The same measures were then taken after the exposure period at follow-up on the same day (two sets of measurements per subject). Blood pressure measurements were collected after at least 5 min of quiet rest using Space Labs Ambulatory Blood Pressure Monitors (MODEL 90217A, Space Labs, Hertford, UK) and endothelial function was measured using an EndoPAT 2000 instrument (Itamar Medical Ltd, Cesari, Israel). For the endothelial function test, participants had a finger clip placed on one finger of each hand and the blood flow occluded to one hand, resulting in occlusion of the brachial artery for 5 min using a standard blood pressure cuff. The surge in blood flow (reactive hyperemia) that occurs when the blood pressure cuff is released results in flow-mediated vasodilation, which is measured by the EndoPAT instrument. Endothelial dysfunction is present if there is attenuation of this dilation. This EF test can cause some discomfort but is otherwise non-invasive. The EndoPAT instrument uses concurrent measurements collected from the digital vasculature of the occluded and non-occluded arms to determine the Reactive Hyperemia Index (RHI).

Heart rate variability (HRV) was measured on one study day for each participant using three-channel (seven lead) digital Holter monitors (SEER Light Extend, GE Medical Systems Information Technologies Inc, Milwaukee, Wi, USA) worn on the body. Both time-domain measures (standard deviation of all normal-to-normal (NN) intervals (SDNN)), the root mean square of successive NN interval differences (RMSSD)) and frequency-domain (low-frequency power (LF), high-frequency power (HF), and low-to-high frequency power ratio (LF:HF)) measures of HRV were examined. RMSSD and HF represent parasympathetic modulation of the heart, SDNN represents total power and LF represents both parasympathetic and sympathetic modulation. The LF:HF ratio was also estimated and represents the balance between sympathetic and parasympathetic modulation^62^. Mean heart rate was also recorded. Participants were instructed to trigger their Holter monitor every 30 min to record repeated measurements (corresponding to 30-min exposure windows), resulting in up to 16 measurements per participant (participants received text messages to remind them to trigger the monitors). The last 5 min of each 30-min exposure window were used to determine HRV parameters, according to guidelines^66^, and experts from the Ottawa Heart Institute analyzed all HRV measures included in the final analysis.

### Statistical analysis

We used multivariable linear mixed-effects models, with random subject intercepts, to assess the associations between daily mean personal and, in separate models, ambient pollutant concentrations and acute changes, from baseline to follow-up measurements during the same day, in endothelial function, blood pressure, heart rate, and HRV parameters adjusted for covariates. Next, we modelled the associations between repeated short-term 30-min mean personal exposures and, in separate models, 30-min mean ambient fixed-site pollutant concentrations on subacute changes in HRV parameters, at the end of the 30-min interval. We included random intercepts and slopes for individuals, with a first order autoregressive structure to account for potential temporal autocorrelations between error terms for consecutive outcome measurements within the same individual. We used transformations for select log-normally distributed exposures and outcomes in models of repeated 30-min mean exposures, using log base 5 for UFP exposure and natural logs for both BC exposures and three outcomes: LF, HF and LF:HF ratio, for which this transformation satisfied the normality assumption. For UFP and BC exposures, we chose transformations such that the increment of increase in the log transformed model would approximately reflect an effect of increasing the untransformed exposure variable by its IQR. All models included a linear term for mean air temperature, averaged over the relevant exposure time window. We also considered previous 24-h caffeine and 24-h alcohol intakes, and relative humidity, as potential confounders. These variables were included in final models if they had a meaningful impact (a change of ≥ 10%) on model coefficients for pollution.

Model coefficients where no transformations were employed reflect mean within-person changes in the outcome for each interquartile range (IQR) change in exposure. Model coefficients where outcomes were natural log transformed reflect a percent change in the outcome per IQR change in exposure. Lastly, model coefficients for log transformed exposures reflect a change in outcome (mean absolute or percent change) associated with a ~ fivefold (UFPs) or a ~ 2.7-fold (BC) increase in exposure. We fit single and two-pollutant models (air pollutants and air pollutants + noise, respectively) to evaluate potential confounding by noise in the air pollutant-outcome associations. We performed regression diagnostics to assess model assumptions, added quadratic exposure terms to assess possible non-linearities, and finally selected the best-fitting models based on the minimum Akaike Information Criterion (AIC). In the repeated measures analyses of HRV, we conducted sensitivity analyses examining possible effect modification of air pollution associations by personal noise exposure, dichotomized at the median (< 68.1 dBA vs ≥ 68.1 dBA), as well as by participants’ sex, by including the appropriate interaction term. If an interaction was detected, we examined stratified models. We also assessed whether or not associations with UFPs, BC and PM_2.5_ were modified by O_x_ dichotomized at the median (< 24.3 ppb vs ≥ 24.3 ppb), as some evidence suggests that O_x_ may modify the cardiovascular health impacts of particulate matter exposure^51,52^.

Finally, we examined flexible weighted cumulative exposure (WCE) mixed-effects models to assess the importance of timing of exposure on changes in two measures of HRV (i.e. SDNN and RMSSD) in response to 30-min exposures. This method models the cumulative effects of past exposures as their weighted sum^67^. Weights, modeled with unpenalized cubic regression B-splines, reflect the relative importance of exposures occurring at different times in the past. Because of the limited sample size, we considered only parsimonious 1-knot spline models, and a priori association restricted the relevant exposure window to the four hours before the outcome measurement, with the weight function constrained to decay to zero at the end of the window. This implied 3 degrees of freedom for the flexible spline estimate. In WCE analyses, we accounted for random intercepts. The main purpose of this analysis was to evaluate how the timing of exposure influences the acute cardiovascular health impacts of personal exposures to UFPs, BC, and noise, and to account for potential short-term cumulative effects of these exposures. Data analysis was conducted using STATA 14 (StataCorp, College Station, TX) and R Statistical software (R-project.org).

## Supplementary information

Supplementary Information.

## Data Availability

Data are available by contacting Dr. Scott Weichenthal at scott.weichenthal@mcgill.ca.

## References

[1] Naghavi M (2017). Global, regional, and national age-sex specific mortality for 264 causes of death, 1980–2016: a systematic analysis for the Global Burden of Disease Study 2016. Lancet (London, England).

[2] 2WHO. *Ambient Air Pollution: A Global Assessment of Exposure and Burden of Disease* (Geneva, Switzerland, 2016).

[3] Munzel T, Gori T, Babisch W, Basner M (2014). Cardiovascular effects of environmental noise exposure. Eur. Heart J..

[4] Munzel T (2018). The adverse effects of environmental noise exposure on oxidative stress and cardiovascular risk. Antioxid. Redox Signal..

[5] Babisch W (2011). Cardiovascular effects of noise. Noise & health.

[6] Sorensen M (2012). Road traffic noise and incident myocardial infarction: a prospective cohort study. PLoS ONE.

[7] Sorensen M (2017). Long-term exposure to road traffic noise and nitrogen dioxide and risk of heart failure: a cohort study. Environ. Health Perspect..

[8] Gan WQ, Davies HW, Koehoorn M, Brauer M (2012). Association of long-term exposure to community noise and traffic-related air pollution with coronary heart disease mortality. Am. J. Epidemiol..

[9] Hoffmann B (2009). Residential traffic exposure and coronary heart disease: results from the Heinz Nixdorf Recall Study. Biomark. Biochem. Indic. Exposure Response Susceptibility Chem..

[10] Weichenthal S (2012). Selected physiological effects of ultrafine particles in acute cardiovascular morbidity. Environ. Res..

[11] Buteau S, Goldberg MS (2016). A structured review of panel studies used to investigate associations between ambient air pollution and heart rate variability. Environ. Res..

[12] Magalhaes S, Baumgartner J, Weichenthal S (2018). Impacts of exposure to black carbon, elemental carbon, and ultrafine particles from indoor and outdoor sources on blood pressure in adults: a review of epidemiological evidence. Environ. Res..

[13] Munzel T (2018). Effects of gaseous and solid constituents of air pollution on endothelial function. Eur. Heart J..

[14] Zuurbier M (2010). Commuters' exposure to particulate matter air pollution is affected by mode of transport, fuel type, and route. Environ. Health Perspect..

[15] Peretz A (2008). Diesel exhaust inhalation elicits acute vasoconstriction in vivo. Environ. Health Perspect..

[16] Pitchika A (2017). Long-term associations of modeled and self-reported measures of exposure to air pollution and noise at residence on prevalent hypertension and blood pressure. Sci. Total Environ..

[17] Zijlema W (2016). Road traffic noise, blood pressure and heart rate: pooled analyses of harmonized data from 88,336 participants. Environ. Res..

[18] Chang LT (2015). Short-term exposure to noise, fine particulate matter and nitrogen oxides on ambulatory blood pressure: a repeated-measure study. Environ. Res..

[19] Roswall N (2017). Long-term residential road traffic noise and NO2 exposure in relation to risk of incident myocardial infarction—a Danish cohort study. Environ. Res..

[20] Tonne C (2016). Long-term traffic air and noise pollution in relation to mortality and hospital readmission among myocardial infarction survivors. Int. J. Hyg. Environ. Health.

[21] Heritier H (2019). A systematic analysis of mutual effects of transportation noise and air pollution exposure on myocardial infarction mortality: a nationwide cohort study in Switzerland. Eur. Heart J..

[22] Boogaard H, Borgman F, Kamminga J, Hoek G (2009). Exposure to ultrafine and fine particles and noise during cycling and driving in 11 Dutch cities. Atmos. Environ..

[23] Sears CG (2018). The association of traffic-related air and noise pollution with maternal blood pressure and hypertensive disorders of pregnancy in the HOME study cohort. Environ Int.

[24] Peters A (2015). Elevated particle number concentrations induce immediate changes in heart rate variability: a panel study in individuals with impaired glucose metabolism or diabetes. Particle Fibre Toxicol..

[25] Hampel R (2014). Impact of personally measured pollutants on cardiac function. Int. J. Hyg. Environ. Health.

[26] Gong J (2014). Comparisons of ultrafine and fine particles in their associations with biomarkers reflecting physiological pathways. Environ. Sci. Technol..

[27] Kubesch N (2015). Arterial blood pressure responses to short-term exposure to low and high traffic-related air pollution with and without moderate physical activity. Eur. J. Prev. Cardiol..

[28] Olsen Y (2014). Vascular and lung function related to ultrafine and fine particles exposure assessed by personal and indoor monitoring: a cross-sectional study. Environ. Health.

[29] Rich DQ (2012). Are ambient ultrafine, accumulation mode, and fine particles associated with adverse cardiac responses in patients undergoing cardiac rehabilitation?. Environ. Health Perspect..

[30] Weichenthal S (2011). Traffic-related air pollution and acute changes in heart rate variability and respiratory function in urban cyclists. Environ. Health Perspect..

[31] Vora R (2014). Inhalation of ultrafine carbon particles alters heart rate and heart rate variability in people with type 2 diabetes. Particle Fibre Toxicol..

[32] Weichenthal S, Hatzopoulou M, Goldberg MS (2014). Exposure to traffic-related air pollution during physical activity and acute changes in blood pressure, autonomic and micro-vascular function in women: a cross-over study. Particle Fibre Toxicol..

[33] Weichenthal S (2012). Personal exposure to specific volatile organic compounds and acute changes in lung function and heart rate variability among urban cyclists. Environ. Res..

[34] Mills NL (2005). Diesel exhaust inhalation causes vascular dysfunction and impaired endogenous fibrinolysis. Circulation.

[35] Lee MS (2016). Effects of personal exposure to ambient fine particulate matter on acute change in nocturnal heart rate variability in subjects without overt heart disease. Am. J. Cardiol..

[36] Tornqvist H (2007). Persistent endothelial dysfunction in humans after diesel exhaust inhalation. Am. J. Respir. Crit. Care Med..

[37] Devlin RB (2014). Controlled exposure of humans with metabolic syndrome to concentrated ultrafine ambient particulate matter causes cardiovascular effects. Toxicol. Sci. Off. J. Soc. Toxicol..

[38] Cole-Hunter T (2016). Impact of traffic-related air pollution on acute changes in cardiac autonomic modulation during rest and physical activity: a cross-over study. J. Eposure Sci. Environ. Epidemiol..

[39] Sarnat JA (2014). Exposure to traffic pollution, acute inflammation and autonomic response in a panel of car commuters. Environ. Res..

[40] Morishita M (2019). Acute blood pressure and cardiovascular effects of near-roadway exposures with and without N95 respirators. Am. J. Hypertens..

[41] Moshammer H (2019). Acute effects of air pollution and noise from road traffic in a panel of young healthy adults. Int. J. Environ. Res. Public Health.

[42] Rundell KW, Hoffman JR, Caviston R, Bulbulian R, Hollenbach AM (2007). Inhalation of ultrafine and fine particulate matter disrupts systemic vascular function. Inhalation Toxicol..

[43] Brook RD (2016). Extreme air pollution conditions adversely affect blood pressure and insulin resistance: the air pollution and cardiometabolic disease study. Hypertension (Dallas, Tx, 1979).

[44] Kraus U (2013). Individual daytime noise exposure during routine activities and heart rate variability in adults: a repeated measures study. Environ. Health Perspect..

[45] Walker ED, Brammer A, Cherniack MG, Laden F, Cavallari JM (2016). Cardiovascular and stress responses to short-term noise exposures-a panel study in healthy males. Environ. Res..

[46] Chang TY, Lai YA, Hsieh HH, Lai JS, Liu CS (2009). Effects of environmental noise exposure on ambulatory blood pressure in young adults. Environ. Res..

[47] Chang TY, Liu CS, Hsieh HH, Bao BY, Lai JS (2012). Effects of environmental noise exposure on 24-h ambulatory vascular properties in adults. Environ. Res..

[48] Devlin RB, Ghio AJ, Kehrl H, Sanders G, Cascio W (2003). Elderly humans exposed to concentrated air pollution particles have decreased heart rate variability. Eur. Respir. J. Suppl..

[49] Cavallari JM (2008). Time course of heart rate variability decline following particulate matter exposures in an occupational cohort. Inhalation Toxicol..

[50] Peters A (2013). Triggering of acute myocardial infarction by different means of transportation. Eur. J. Prev. Cardiol..

[51] Lavigne E, Burnett RT, Weichenthal S (2018). Association of short-term exposure to fine particulate air pollution and mortality: effect modification by oxidant gases. Sci. Rep..

[52] Weichenthal S, Pinault LL, Burnett RT (2017). Impact of oxidant gases on the relationship between outdoor fine particulate air pollution and nonaccidental, cardiovascular, and respiratory mortality. Sci. Rep..

[53] Daiber A (2019). Environmental noise induces the release of stress hormones and inflammatory signaling molecules leading to oxidative stress and vascular dysfunction-Signatures of the internal exposome. BioFactors (Oxf., Engl.).

[54] Brook RD (2008). Cardiovascular effects of air pollution. Clin. Sci. (Lond., Engl).

[55] Laumbach RJ (2014). A controlled trial of acute effects of human exposure to traffic particles on pulmonary oxidative stress and heart rate variability. Particle Fibre Toxicol..

[56] Wessels A (2010). Oxidant generation and toxicity of size-fractionated ambient particles in human lung epithelial cells. Environ. Sci. Technol..

[57] Kinlay S, Ganz P (1997). Role of endothelial dysfunction in coronary artery disease and implications for therapy. Am. J. Cardiol..

[58] Anderson TJ (1999). Assessment and treatment of endothelial dysfunction in humans. J. Am. Coll. Cardiol..

[59] Ikeda M (1998). Mechanism of pathophysiological effects of diesel exhaust particles on endothelial cells. Environ. Toxicol. Pharmacol..

[60] Lee MS (2014). Oxidative stress and systemic inflammation as modifiers of cardiac autonomic responses to particulate air pollution. Int. J. Cardiol..

[61] Stieb DM (2017). Cardio-respiratory effects of air pollution in a panel study of outdoor physical activity and health in rural older adults. J. Occup. Environ. Med..

[62] Stein PK, Kleiger RE (1999). Insights from the study of heart rate variability. Annu. Rev. Med..

[63] Weisskopf MG, Seals RM, Webster TF (2018). Bias amplification in epidemiologic analysis of exposure to mixtures. Environ. Health Perspect..

[64] Weichenthal S, Lavigne E, Evans G, Pollitt K, Burnett RT (2016). Ambient PM2.5 and risk of emergency room visits for myocardial infarction: impact of regional PM2.5 oxidative potential: a case-crossover study. Environ. Health.

[65] Bratsch SG (1989). Standard electrode potentials and temperature coefficients in water. J. Phys. Chem. Ref. Data.

[66] Camm AJ (1996). Heart rate variability: standards of measurement, physiological interpretation and clinical use. Task force of the European Society of Cardiology and the North American Society of Pacing and Electrophysiology. Circulation.

[67] Danieli, C., Sheppard, T., Costello, R., Dixon, W. G. & Abrahamowicz, M. Modeling of cumulative effects of time-varying drug exposures on within-subject changes in a continuous outcome. *Stat. Methods Med. Res. 0*, 0962280220902179, doi:10.1177/0962280220902179. 10.1177/0962280220902179 32020828

[68] Douketis JD, Paradis G, Keller H, Martineau C (2005). Canadian guidelines for body weight classification in adults: application in clinical practice to screen for overweight and obesity and to assess disease risk. Can. Med. Assoc. J..

