# Supplementary material for: Acute cardiovascular health effects in a panel study of personal exposure to traffic-related air pollutants and noise in Toronto, Canada
Source: Sci Rep. 2020 Oct 7;10:16703. doi: 10.1038/s41598-020-73412-6 (PMC7541521; doi:10.1038/s41598-020-73412-6)

**Supplementary Information**

**Acute cardiovascular health effects in a panel study of personal exposure to traffic-related air pollutants and noise in Toronto, Canada**

Rita Biel, Coraline Danieli, Maryam Shekarrizfard, Laura Minet, Michal Abrahamowicz, Jill Baumgartner, Rick Liu, Marianne Hatzopoulou, Scott Weichenthal

**Supplementary Table S1. Distribution of baseline physiological measures (data for both study visits) ^^^**

| **Physiological measure** | **n** | **Mean (sd)** | **Range** |
| --- | --- | --- | --- |
| Heart rate (bpm) ^*^ | 87 | 69.9 (11.5) | 47.7-102.7 |
| Heart rate (bpm) Holter ^*^ | 45 | 70.7 (11.7) | 46-98 |
| Systolic blood pressure (mmHg) ^**^ | 87 | 114.1 (11.6) | 91-138.7 |
| Diastolic blood pressure (mmHg) ^**^ | 87 | 64.9 (7.9) | 51-84.3 |
| Reactive Hyperemia Index | 85 | 2.06 (0.73) | 0.88-4.1 |
| SDNN (ms) | 45 | 70.9 (28.4) | 19-149 |
| RMSSD (ms) | 45 | 48.9 (26.7) | 8.0-116.0 |
| LF (ms^2^) | 45 | 1,131.7 (1,198.0) | 26.6-6,075.3 |
| HF (ms^2^) | 45 | 866.6 (853.4) | 11.4-3,543.2 |
| LF:HF | 45 | 2.04 (1.69) | 0.5-7.2 |
| ^^^ Data reflect 2 visits per person, except for 5 participants who did not do visit 2. 1 participant had missing HRV measures on visit 1. ^*^Heart rate was measured by the Holter monitor at baseline and follow-up during visit 1 when participants wore a Holter monitor for HRV measures. ^*^Heart rate was also measured by a blood pressure monitor on both visits, 3 times at baseline and 3 times at follow-up, where each final measure was a mean of 3 readings. ^**^Measure was a mean of 3 readings. | | | |

**Supplementary Table S2. Pearson correlation coefficients for distribution of personal and regional fixed-site daily average and 30-minute average pollutant exposures and environmental variables.**

| **Exposures by time window** | UFPs (particles/cm^3^) | Black carbon (ng/m^3^) | Noise (dBA) | PM_2.5_ (µg/m^3^) | NO_2_ (ppb) | O_3_ (ppb) | O_x_ (ppb) | Temperature (˚C) | RH (%) |
| --- | --- | --- | --- | --- | --- | --- | --- | --- | --- |
| **Daily average (both study visits)** | | | | | | | | | |
| UFPs (particles/cm^3^) | 1.0 | . | . | . | . | . | . | . | . |
| Black carbon (ng/m^3^) | -0.005 | 1.0 | . | . | . | . | . | . | . |
| Noise (dBA) | 0.270 | 0.392 | 1.0 | . | . | . | . | . | . |
| PM_2.5_ (µg/m^3^) | -0.111 | 0.343 | 0.040 | 1.0 | . | . | . | . | . |
| NO_2_ (ppb) | 0.003 | 0.157 | -0.049 | 0.347 | 1.0 | . | . | . | . |
| O_3_ (ppb) | -0.109 | 0.081 | 0.042 | **0.650** | -0.077 | 1.0 | . | . | . |
| O_x_ (ppb) | -0.107 | 0.117 | 0.031 | **0.722** | 0.144 | **0.976** | 1.0 | . | . |
| Temperature (˚C) | 0.037 | 0.305 | 0.027 | **0.704** | 0.181 | 0.327 | 0.364 | 1.0 | . |
| RH (%) | -0.022 | 0.225 | 0.152 | 0.269 | **-0.512** | 0.196 | 0.082 | 0.286 | 1.0 |
| **30-minute average (first study visit)** | | | | | | | | | |
| UFPs (particles/cm^3^) | 1.0 | . | . | . | . | . | . | . | . |
| Black carbon (ng/m^3^) | 0.037 | 1.0 | . | . | . | . | . | . | . |
| Noise (dBA) | 0.121 | 0.301 | 1.0 | . | . | . | . | . | . |
| PM_2.5_ (µg/m^3^) | 0.001 | 0.180 | 0.024 | 1.0 | . | . | . | . | . |
| NO_2_ (ppb) | 0.005 | 0.076 | 0.098 | 0.141 | 1.0 | . | . | . | . |
| O_3_ (ppb) | -0.007 | 0.107 | -0.070 | **0.454** | -0.342 | 1.0 | . | . | . |
| O_x_ (ppb) | -0.007 | 0.124 | -0.058 | **0.497** | -0.193 | **0.988** | 1.0 | . | . |
| Temperature (˚C) | 0.066 | 0.131 | -0.025 | **0.445** | -0.123 | **0.530** | **0.533** | 1.0 | . |
| RH (%) | -0.019 | 0.070 | 0.060 | **0.500** | -0.003 | -0.112 | -0.118 | -0.57 | 1.0 |

**Supplementary Table S3. Associations between daily average pollutant measurements and baseline to follow-up changes in Reactive Hyperemia Index (RHI), blood pressure (BP) and heart rate, in single pollutant models.**

|  | | **Temperature-adjusted model^^^** | | | | **Fully-adjusted model^^^^** | | |
| --- | --- | --- | --- | --- | --- | --- | --- | --- |
| **Outcome** | **Exposure** | **IQR** | **n/N** | **Coefficient** | **95% Cl** | **n/N** | **Coefficient** | **95% CI** |
| **RHI** | **Personal exposures** |  |  |  |  |  |  |  |
|  | UFPs (particles/cm^3^) | 14,890 | 78/46 | 0.103 | -0.04, 0.24 | 76/46 | 0.101 | -0.04, 0.24 |
|  | Black carbon (ng/m^3^) | 1,464 | 81/46 | 0.048 | -0.12, 0.21 | 79/46 | 0.021 | -0.14, 0.18 |
|  | Noise (dBA) | 5.2 | 79/44 | 0.031 | -0.18, 0.24 | 77/44 | 0.051 | -0.16, 0.26 |
|  | **Fixed-site exposures** |  |  |  |  |  |  |  |
|  | PM_2.5_ (µg/m^3^) | 5.5 | 84/46 | **-0.384** | **-0.73, -0.04** | 82/46 | **-0.452** | **-0.81**, **-0.09** |
|  | NO_2_ (ppb) | 2.7 | 84/46 | -0.046 | -0.18, 0.09 | 82/46 | -0.061 | -0.20, 0.07 |
|  | O_3_ (ppb) | 12.2 | 84/46 | **-0.269** | **-0.54**, **-0.003** | 82/46 | **-0.292** | **-0.56**, **-0.02** |
|  | O_x_ (ppb) | 7.4 | 84/46 | **-0.276** | **-0.52**, **-0.03** | 82/46 | **-0.311** | **-0.56**, **-0.06** |
|  |  |  |  |  |  |  |  |  |
| **Systolic** | **Personal exposures** |  |  |  |  |  |  |  |
| **BP** | UFPs (particles/cm^3^) | 14,890 | 81/46 | 0.521 | -1.00, 2.04 | 79/46 | 0.417 | -1.10, 1.94 |
|  | Black carbon (ng/m^3^) | 1,464 | 84/46 | **-2.94** | **-4.80**, **-1.07** | 82/46 | **-2.86** | **-4.78**, **-0.94** |
|  | Noise (dBA) | 5.2 | 82/45 | -1.05 | -3.18, 1.09 | 80/45 | -0.99 | -3.16, 1.18 |
|  | **Fixed-site exposures^*^** |  |  |  |  |  |  |  |
|  | PM_2.5_ (µg/m^3^) | 5.5 | 87/46 | -1.61 | -5.90, 2.68 | 85/46 | -0.931 | -5.44, 3.58 |
|  | NO_2_ (ppb) | 2.7 | 87/46 | 0.509 | -1.13, 2.15 | 85/46 | 0.653 | -1.00, 2.31 |
|  | O_3_ (ppb) | 12.2 | 87/46 | 0.085 | -3.21, 3.38 | 85/46 | 0.636 | -2.73, 4.01 |
|  | O_x_ (ppb) | 7.4 | 87/46 | 0.307 | -2.75, 3.37 | 85/46 | 0.900 | -2.25, 4.04 |
|  |  |  |  |  |  |  |  |  |
| **Diastolic** | **Personal exposures** |  |  |  |  |  |  |  |
| **BP** | UFPs (particles/cm^3^) | 14,890 | 81/46 | 0.433 | -0.59, 1.46 | 79/46 | 0.347 | -0.67, 1.37 |
|  | Black carbon (ng/m^3^) | 1,464 | 84/46 | -0.97 | -2.29, 0.35 | 82/46 | **-1.13** | **-2.47, 0.21** |
|  | Noise (dBA) | 5.2 | 82/45 | -0.048 | -1.74, 1.64 | 80/45 | -0.101 | -1.82, 1.62 |
|  | **Fixed-site exposures** |  |  |  |  |  |  |  |
|  | PM_2.5_ (µg/m^3^) | 5.5 | 87/46 | 1.78 | -1.07, 4.64 | 85/46 | **2.45** | **-0.53**, **5.44** |
|  | NO_2_ (ppb) | 2.7 | 87/46 | -0.220 | -1.32, 0.89 | 85/46 | -0.160 | -1.27, 0.95 |
|  | O_3_ (ppb) | 12.2 | 87/46 | **1.28** | **-0.91, 3.47** | 85/46 | **1.67** | **-0.56**, **3.91** |
|  | O_x_ (ppb) | 7.4 | 87/46 | **1.10** | **-0.93, 3.14** | 85/46 | **1.52** | **-0.58**, **3.61** |
|  |  |  |  |  |  |  |  |  |
| **Heart** | **Personal exposures** |  |  |  |  |  |  |  |
| **Rate^*^** | UFPs (particles/cm^3^) | 14,890 | 81/46 | 0.89 | -0.48, 2.27 | 79/46 | 0.90 | -0.47, 2.26 |
|  | Black carbon (ng/m^3^) | 1,464 | 84/46 | -0.45 | -2.33, 1.42 | 82/46 | -0.59 | -2.45, 1.28 |
|  | Noise (dBA) | 5.2 | 82/45 | 0.19 | -2.20, 2.59 | 80/45 | 0.092 | -2.30, 2.49 |
|  | **Fixed-site exposures** |  |  |  |  |  |  |  |
|  | PM_2.5_ (µg/m^3^) | 5.5 | 87/46 | 0.712 | -3.32, 4.74 | 85/46 | 0.265 | -3.95, 4.48 |
|  | NO_2_ (ppb) | 2.7 | 87/46 | -0.445 | -1.88, 0.98 | 85/46 | -0.311 | -1.73, 1.11 |
|  | O_3_ (ppb) | 12.2 | 87/46 | 1.17 | -1.90, 4.25 | 85/46 | 1.22 | -1.84, 4.29 |
|  | O_x_ (ppb) | 7.4 | 87/46 | 0.894 | -1.99, 3.78 | 85/46 | 1.02 | -1.86, 3.91 |
| UFPs - ultrafine particles. IQR - interquartile range. Regression coefficients reflect the absolute change per IQR increase in exposure.  ^*^Heart rate measured by a blood pressure monitor on both visits, 3 times at baseline and 3 times at follow-up, where the final measure was a mean of these 3 readings. ^^^ adjusted for continuous temperature (degrees Celsius).  ^^^^ adjusted for continuous temperature (degrees Celsius), alcohol intake (yes/no) and caffeine intake (yes/no) in the last 24 hours. | | | | | | | | |

**Supplementary Table S4. Associations between daily average pollutant measurements and baseline to follow-up changes in HRV parameters, in single-pollutant models.**

|  | | **Temperature-adjusted model^^^** | | | | **Fully-adjusted model^^^^** | | |
| --- | --- | --- | --- | --- | --- | --- | --- | --- |
| **Outcome** | **Exposure** | **IQR** | **n/N ^*^** | **Coefficient** | **95% Cl** | **n/N ^*^** | **Coefficient** | **95% CI** |
| **SDNN** | **Personal exposures** |  |  |  |  |  |  |  |
|  | UFPs (particles/cm^3^) | 14,890 | 42/42 | -3.71 | -11.5, 4.12 | 40/40 | -4.55 | -12.4, 3.34 |
|  | Black carbon (ng/m^3^) | 1,464 | 44/44 | **-9.25** | **-19.1**, **-0.55** | 42/42 | **-8.31** | **-19.1, 2.49** |
|  | Noise (dBA) | 5.2 | 41/41 | -10.0 | -23.7, 3.66 | 39/39 | -11.7 | -26.8, 3.33 |
|  | **Fixed-site exposures^*^** |  |  |  |  |  |  |  |
|  | PM_2.5_ (µg/m^3^) | 5.5 | 45/45 | -4.18 | -26.5, 18.1 | 43/43 | 4.96 | -20.7, 30.7 |
|  | NO_2_ (ppb) | 2.7 | 45/45 | 3.38 | -4.03, 10.8 | 43/43 | 4.12 | -3.41, 11.6 |
|  | O_3_ (ppb) | 12.2 | 45/45 | 1.69 | -16.3, 19.6 | 43/43 | 4.50 | -14.3, 23.3 |
|  | O_x_ (ppb) | 7.4 | 45/45 | 3.68 | -12.9, 20.3 | 43/43 | 7.07 | -10.5, 24.6 |
|  |  |  |  |  |  |  |  |  |
| **RMSSD** | **Personal exposures** |  |  |  |  |  |  |  |
|  | UFPs (particles/cm^3^) | 14,890 | 42/42 | **-4.92** | **-9.21, -0.63** | 40/40 | **-5.10** | **-9.62, -0.58** |
|  | Black carbon (ng/m^3^) | 1,464 | 44/44 | -3.80 | -9.77, 2.17 | 42/42 | -3.73 | -10.5, 3.05 |
|  | Noise (dBA) | 5.2 | 41/41 | **-7.03** | **-14.9, 0.83** | 39/39 | **-7.91** | **-16.8, 1.01** |
|  | **Fixed-site exposures^*^** |  |  |  |  |  |  |  |
|  | PM_2.5_ (µg/m^3^) | 5.5 | 45/45 | 6.77 | -6.26, 19.8 | 43/43 | 10.8 | -4.55, 26.2 |
|  | NO_2_ (ppb) | 2.7 | 45/45 | 2.28 | -2.10, 6.65 | 43/43 | 2.37 | -2.24, 6.98 |
|  | O_3_ (ppb) | 12.2 | 45/45 | 0.06 | -10.6, 10.7 | 43/43 | 0.36 | -11.2, 11.9 |
|  | O_x_ (ppb) | 7.4 | 45/45 | 1.47 | -8.37, 11.3 | 43/43 | 1.94 | -8.88, 12.8 |
|  |  |  |  |  |  |  |  |  |
| **LF** | **Personal exposures** |  |  |  |  |  |  |  |
|  | UFPs (particles/cm^3^) | 14,890 | 42/42 | -142.9 | -377.0, 91.2 | 40/40 | -162.5 | -404.7, 80.6 |
|  | Black carbon (ng/m^3^) | 1,464 | 44/44 | 44.5 | -252.6, 341.6 | 42/42 | 9.00 | -321.0, 339.0 |
|  | Noise (dBA) | 5.2 | 41/41 | -33.9 | -448.9, 381.1 | 39/39 | -45.3 | -511.5, 420.9 |
|  | **Fixed-site exposures^*^** |  |  |  |  |  |  |  |
|  | PM_2.5_ (µg/m^3^) | 5.5 | 45/45 | 118.7 | -524.3, 761.7 | 43/43 | 142.6 | -609.4, 894.6 |
|  | NO_2_ (ppb) | 2.7 | 45/45 | 81.6 | -133.1, 296.2 | 43/43 | 94.4 | -126.9, 315.7 |
|  | O_3_ (ppb) | 12.2 | 45/45 | 50.2 | -467.8, 568.2 | 43/43 | 22.8 | -529.0, 574.6 |
|  | O_x_ (ppb) | 7.4 | 45/45 | 97.4 | -382.1, 576.9 | 43/43 | 85.4 | -431.6, 602.4 |
|  |  |  |  |  |  |  |  |  |
| **HF** | **Personal exposures** |  |  |  |  |  |  |  |
|  | UFPs (particles/cm^3^) | 14,890 | 42/42 | -124.4 | -289.7, 40.9 | 40/40 | **-123.5** | **-293.1, 46.0** |
|  | Black carbon (ng/m^3^) | 1,464 | 44/44 | -131.4 | -337.6, 74.7 | 42/42 | -99.4 | -328.9, 130.2 |
|  | Noise (dBA) | 5.2 | 41/41 | -173.6 | -460.0, 107.3 | 39/39 | **-165.2** | **-480.2, 149.7** |
|  | **Fixed-site exposures** |  |  |  |  |  |  |  |
|  | PM_2.5_ (µg/m^3^) | 5.5 | 45/45 | 64.0 | -391.1, 519.0 | 43/43 | 169.6 | -357.0, 696.2 |
|  | NO_2_ (ppb) | 2.7 | 45/45 | -22.8 | -175.5, 129.9 | 43/43 | -28.2 | -185.0, 128.6 |
|  | O_3_ (ppb) | 12.2 | 45/45 | -75.6 | -441.3, 290.2 | 43/43 | -58.9 | -446.3, 328.5 |
|  | O_x_ (ppb) | 7.4 | 45/45 | -84.6 | -423.4, 254.1 | 43/43 | -75.2 | -438.3, 287.8 |
|  |  |  |  |  |  |  |  |  |
| **LF:HF** | **Personal exposures** |  |  |  |  |  |  |  |
|  | UFPs (particles/cm^3^) | 14,890 | 42/42 | 0.19 | -0.39, 0.77 | 40/40 | 0.14 | -0.44, 0.72 |
|  | Black carbon (ng/m^3^) | 1,464 | 44/44 | -0.12 | -0.93, 0.70 | 42/42 | -0.13 | -1.00, 0.75 |
|  | Noise (dBA) | 5.2 | 41/41 | 0.17 | -1.00, 1.34 | 39/39 | 0.34 | -0.92, 1.59 |
|  | **Fixed-site exposures** |  |  |  |  |  |  |  |
|  | PM_2.5_ (µg/m^3^) | 5.5 | 45/45 | **-1.72** | **-3.40**, **0.04** | 43/43 | **-1.77** | **-3.68, 0.14** |
|  | NO_2_ (ppb) | 2.7 | 45/45 | -0.26 | -0.85, 0.32 | 43/43 | -0.20 | -0.79, 0.39 |
|  | O_3_ (ppb) | 12.2 | 45/45 | **-1.15** | **-2.52, 0.22** | 43/43 | **-1.06** | **-2.49, 0.35** |
|  | O_x_ (ppb) | 7.4 | 45/45 | **-1.24** | **-2.5**, **0.02** | 43/43 | **-1.15** | **-2.47, 0.17** |
| UFPs - ultrafine particles. IQR - interquartile range. Regression coefficients reflect the absolute change per IQR increase in exposure. ^^^ adjusted for continuous temperature (degrees Celsius). ^^^^ adjusted for continuous temperature (degrees Celsius), alcohol intake (yes/no) and caffeine intake (yes/no) in the last 24 hours. | | | | | | | | |

**Supplementary Table S5. Associations between daily average pollutant measurements and baseline to follow-up changes in Reactive Hyperemia Index (RHI), blood pressure (BP) and heart rate, in two-pollutant models (air pollutant + noise).**

|  | | **Temperature-adjusted model^^^** | | | | **Fully-adjusted model^^^^** | | |
| --- | --- | --- | --- | --- | --- | --- | --- | --- |
| **Outcome** | **Exposure** | **IQR** | **n/N^*^** | **Coefficient** | **95% Cl** | **n/N ^*^** | **Coefficient** | **95% CI** |
| **RHI** | **Personal exposures** |  |  |  |  |  |  |  |
|  | UFPs (particles/cm^3^) | 14,890 | 73/44 | **0.14** | **-0.02, 0.30** | 71/44 | **0.14** | **-0.02, 0.29** |
|  | Noise (dBA) | 5.2 |  | 0.004 | -0.23, 0.24 |  | 0.06 | -0.17, 0.29 |
|  | Black carbon (ng/m^3^) | 1,464 | 76/43 | 0.11 | -0.14, 0.36 | 74/43 | -0.038 | -0.22, 0.14 |
|  | Noise (dBA) | 5.2 |  | 0.02 | -0.03, 0.07 |  | 0.18 | -0.07, 0.44 |
|  | **Fixed-site exposures** |  |  |  |  |  |  |  |
|  | PM_2.5_ (µg/m^3^) | 5.5 | 79/44 | **-0.40** | **-0.76, -0.04** | 77/44 | **-0.47** | **-0.85, -0.09** |
|  | Noise (dBA) | 5.2 |  | 0.05 | -0.16, 0.25 |  | 0.07 | -0.13, 0.28 |
|  | NO_2_ (ppb) | 2.7 | 79/44 | -0.036 | -0.17, 0.10 | 77/44 | -0.051 | -0.18, 0.08 |
|  | Noise (dBA) | 5.2 |  | 0.031 | -0.18, 0.24 |  | 0.05 | -0.16, 0.26 |
|  | O_3_ (ppb) | 12.2 | 79/44 | **-0.27** | **-0.55, 0.003** | 77/44 | **-0.29** | **-0.56, 0.01** |
|  | Noise (dBA) | 5.2 |  | 0.05 | -0.16, 0.25 |  | 0.07 | -0.13, 0.28 |
|  | O_x_ (ppb) | 7.4 | 79/44 | **-0.27** | **-0.53, -0.02** | 77/44 | **-0.30** | **-0.56, -0.04** |
|  | Noise (dBA) | 5.2 |  | 0.05 | -0.16, 0.25 |  | 0.08 | -0.13, 0.28 |
|  |  |  |  |  |  |  |  |  |
| **Systolic** | **Personal exposures** |  |  |  |  |  |  |  |
| **BP** | UFPs (particles/cm^3^) | 14,890 | 76/45 | 1.06 | -0.41, 2.53 | 74/45 | 0.91 | -0.55, 2.37 |
|  | Noise (dBA) | 5.2 |  | -1.78 | -4.10, 0.54 |  | -1.59 | -3.96, 0.78 |
|  | Black carbon (ng/m^3^) | 1,464 | 79/44 | **-2.53** | **-4.33, -0.72** | 77/44 | **-2.47** | **-4.33, -0.62** |
|  | Noise (dBA) | 5.2 |  | -0.09 | -2.55, 2.36 |  | 0.04 | -2.48, 2.56 |
|  | **Fixed-site exposures** |  |  |  |  |  |  |  |
|  | PM_2.5_ (µg/m^3^) | 5.5 | 82/45 | -1.16 | -5.0, 2.67 | 80/45 | -0.63 | -4.66, 3.41 |
|  | Noise (dBA) | 5.2 |  | -1.02 | -3.16, 1.10 |  | -0.19 | -3.14, 1.19 |
|  | NO_2_ (ppb) | 2.7 | 82/45 | 0.43 | -1.01, 1.87 | 80/45 | 0.58 | -0.87, 2.02 |
|  | Noise (dBA) | 5.2 |  | -1.01 | -3.15, 1.12 |  | -0.18 | -3.11, 1.21 |
|  | O_3_ (ppb) | 12.2 | 82/45 | -0.75 | -3.64, 2.14 | 80/45 | -0.32 | -3.31, 2.66 |
|  | Noise (dBA) | 5.2 |  | -1.03 | -3.16, 1.10 |  | -0.98 | -3.14, 1.19 |
|  | O_x_ (ppb) | 7.4 | 82/45 | -0.52 | -3.20, 2.17 | 80/45 | -0.04 | -2.84, 2.76 |
|  | Noise | 5.2 |  | -1.04 | -3.17, 1.10 |  | -0.99 | -3.16, 1.18 |
|  |  |  |  |  |  |  |  |  |
| **Diastolic** | **Personal exposures** |  |  |  |  |  |  |  |
| **BP** | UFPs (particles/cm^3^) | 14,890 | 76/45 | 0.57 | -0.62, 1.76 | 74/45 | 0.50 | -0.69, 1.69 |
|  | Noise (dBA) | 5.2 |  | -0.42 | -2.30, 1.46 |  | -0.37 | -2.29, 1.55 |
|  | Black carbon (ng/m^3^) | 1,464 | 79/44 | **-1.10** | **-2.58, 0.39** | 77/44 | **-1.30** | **-2.81, 0.21** |
|  | Noise (dBA) | 5.2 |  | 0.40 | -1.59, 2.39 |  | 0.51 | -1.52, 2.54 |
|  | **Fixed-site exposures** |  |  |  |  |  |  |  |
|  | PM_2.5_ (µg/m^3^) | 5.5 | 82/45 | 1.83 | -1.18, 4.85 | 80/45 | 2.58 | -0.58, 5.73 |
|  | Noise (dBA) | 5.2 |  | -0.08 | -1.76, 1.60 |  | -0.16 | -1.85, 1.54 |
|  | NO_2_ (ppb) | 2.7 | 82/45 | -0.24 | -1.38, 0.90 | 80/45 | -0.16 | -1.31, 0.99 |
|  | Noise (dBA) | 5.2 |  | -0.07 | -1.76, 1.62 |  | -0.11 | -1.83, 1.61 |
|  | O_3_ (ppb) | 12.2 | 82/45 | 1.28 | -1.00, 3.56 | 80/45 | **1.75** | **-0.58, 4.09** |
|  | Noise (dBA) | 5.2 |  | -0.08 | -1.76, 1.60 |  | -0.15 | -1.85, 1.55 |
|  | O_x_ (ppb) | 7.4 | 82/45 | 1.09 | -1.03, 3.21 | 80/45 | **1.60** | **-0.59, 3.79** |
|  | Noise (dBA) | 5.2 |  | -0.07 | -1.75, 1.61 |  | -0.13 | -1.83, 1.56 |
|  |  |  |  |  |  |  |  |  |
| **Heart** | **Personal exposures** |  |  |  |  |  |  |  |
| **Rate**^**^ | UFPs (particles/cm^3^) | 14,890 | 76/45 | 0.88 | -0.65, 2.42 | 74/45 | 0.91 | -0.61, 2.43 |
|  | Noise (dBA) | 5.2 |  | -1.30 | -3.74,1.15 |  | -1.52 | -3.99, 0.96 |
|  | Black carbon (ng/m^3^) | 1,464 | 79/44 | -0.41 | -2.38, 1.57 | 77/44 | -0.48 | -2.46, 1.49 |
|  | Noise (dBA) | 5.2 |  | -0.89 | -3.76, 1.98 |  | -1.10 | -4.02, 1.81 |
|  | **Fixed-site exposures** |  |  |  |  |  |  |  |
|  | PM_2.5_ (µg/m^3^) | 5.5 | 82/45 | -0.03 | -4.25, 4.20 | 80/45 | -0.57 | -5.02, 3.88 |
|  | Noise (dBA) | 5.2 |  | 0.19 | -2.20, 2.59 |  | 0.13 | -2.28, 2.53 |
|  | NO_2_ (ppb) | 2.7 | 82/45 | -0.52 | -1.97, 0.92 | 80/45 | -0.40 | -1.84, 1.04 |
|  | Noise (dBA) | 5.2 |  | 0.19 | -2.20, 2.58 |  | 0.10 | -2.29, 2.50 |
|  | O_3_ (ppb) | 12.2 | 82/45 | 1.08 | -2.08, 4.24 | 80/45 | 1.16 | -2.01, 4.33 |
|  | Noise (dBA) | 5.2 |  | 0.16 | -2.22, 2.55 |  | 0.04 | -2.35, 2.43 |
|  | O_x_ (ppb) | 7.4 | 82/45 | 0.75 | -2.23, 3.73 | 80/45 | 0.90 | -2.09, 3.90 |
|  | Noise (dBA) | 5.2 |  | 0.17 | -2.21, 2.56 |  | 0.05 | -2.34, 2.43 |
| UFPs - ultrafine particles. IQR - interquartile range. Regression coefficients reflect the absolute change per IQR increase in exposure. ^*^n/N = visits/participants. ^**^Heart rate measured by a blood pressure monitor on both visits, 3 times at baseline and 3 times at follow-up, where the final measure was a mean of these 3 readings. ^^^ adjusted for continuous temperature (degrees Celsius). ^^^^ adjusted for continuous temperature (degrees Celsius), alcohol intake (yes/no) and caffeine intake (yes/no) in the last 24 hours. | | | | | | | | |

**Supplementary Table S6. Associations between daily average pollutant measurements and baseline to follow-up changes in HRV parameters, in two-pollutant models (air pollutant + noise).**

|  | | **Temperature-adjusted model^^^** | | | | **Fully-adjusted model^^^^** | | | |
| --- | --- | --- | --- | --- | --- | --- | --- | --- | --- |
| **Outcome** | **Exposure** | **IQR** | **n/N ^*^** | **Coefficient** | **95% Cl** | **n/N ^*^** | **Coefficient** | | **95% CI** |
| **SDNN** | **Personal exposures** |  |  |  |  |  |  | |  |
|  | UFPs (particles/cm^3^) | 14,890 | 38/38 | **-9.82** | **-19.7, 0.08** | 36/36 | **-10.3** | | **-20.3, 0.35** |
|  | Noise (dBA) | 5.2 |  | -4.26 | -18.4, 9.89 |  | -5.11 | | -21.3, 11.06 |
|  | Black carbon (ng/m^3^) | 1,464 | 40/40 | **-7.71** | **-19.2-3.78** | 38/38 | -6.70 | | -19.0, 5.6 |
|  | Noise (dBA) | 5.2 |  | -3.83 | -21.0, 13.3 |  | -6.76 | | -26.0, 12.5 |
|  | **Fixed-site exposures** |  |  |  |  |  |  | |  |
|  | PM_2.5_ (µg/m^3^) | 5.5 | 41/41 | -6.05 | -29.1-17.0 | 39/39 | -2.28 | | -29.6, 25.0 |
|  | Noise (dBA) | 5.2 |  | -10.5 | -24.5, 3.42 |  | -12.0 | | -27.8, 3.71 |
|  | NO_2_ (ppb) | 2.7 | 41/41 | 2.14 | -5.31-9.59 | 39/39 | 2.57 | | -5.11, 10.2 |
|  | Noise (dBA) | 5.2 |  | -9.61 | -23.5, 4.29 |  | -11.2 | | -26.4, 4.13 |
|  | O_3_ (ppb) | 12.2 | 41/41 | -0.55 | -18.8-17.7 | 39/39 | 0.31 | | -19.4-20.0 |
|  | Noise (dBA) | 5.2 |  | -10.1 | -24.0, 3.88 |  | -11.7 | | -27.2, 3.82 |
|  | O_x_ (ppb) | 7.4 | 41/41 | 0.86 | -16.1-17.8 | 39/39 | 2.19 | | -16.5-20.9 |
|  | Noise (dBA) | 5.2 |  | -9.94 | -23.9, 4.06 |  | -11.4 | | -27.0, 4.25 |
|  |  |  |  |  |  |  |  | |  |
| **RMSSD** | **Personal exposures** |  |  |  |  |  |  | |  |
|  | UFPs (particles/cm^3^) | 14,890 | 38/38 | -3.73 | -9.33, 1.87 | 36/36 | -3.84 | | -9.73, 2.04 |
|  | Noise (dBA) | 5.2 |  | -4.76 | -12.8, 3.24 |  | -5.58 | | -15.1, 3.96 |
|  | Black carbon (ng/m^3^) | 1,464 | 40/40 | -1.45 | -8.20, 5.29 | 38/38 | -1.21 | | -8.61, 6.20 |
|  | Noise (dBA) | 5.2 |  | -6.51 | -16.6, 3.55 |  | -8.22 | | -19.8, 3.34 |
|  | **Fixed-site exposures** |  |  |  |  |  |  | |  |
|  | PM_2.5_ (µg/m^3^) | 5.5 | 41/41 | 7.12 | -5.97, 20.2 | 39/39 | 10.4 | | -5.38, 26.1 |
|  | Noise (dBA) | 5.2 |  | -6.43 | -14.4, 1.50 |  | -6.47 | | -15.6, 2.62 |
|  | NO_2_ (ppb) | 2.7 | 41/41 | 1.56 | -2.40, 6.12 | 39/39 | 1.94 | | -2.59, 6.46 |
|  | Noise (dBA) | 5.2 |  | -6.67 | -14.6, 1.28 |  | -7.48 | | -16.5, 1.5 |
|  | O_3_ (ppb) | 12.2 | 41/41 | 1.29 | -9.19, 11.8 | 39/39 | 1.12 | | -10.5, 12.8 |
|  | Noise (dBA) | 5.2 |  | -6.94 | -15.0, 1.07 |  | -7.77 | | -16.9, 1.42 |
|  | O_x_ (ppb) | 7.4 | 41/41 | 2.40 | -7.31, 12.1 | 39/39 | 2.52 | | -8.52, 13.57 |
|  | Noise (dBA) | 5.2 |  | -6.78 | -14.8, 1.24 |  | -7.49 | | -16.7, 1.73 |
|  |  |  |  |  |  |  |  | |  |
| **LF** | **Personal exposures** |  |  |  |  |  |  | |  |
|  | UFPs (particles/cm^3^) | 14,890 | 38/38 | -230.5 | -547.9, 86.8 | 36/36 | **-236.4** | | **-568.5, 95.7** |
|  | Noise (dBA) | 5.2 |  | 70.8 | -379.4, 521.1 |  | 74.4 | | -464.0, 612.8 |
|  | Black carbon (ng/m^3^) | 1,464 | 40/40 | 114.3 | -240.6, 469.1 | 38/38 | 49.7 | | -338.7, 438.0 |
|  | Noise (dBA) | 5.2 |  | -138.7 | -667.7, 390.2 |  | -98.8 | | -705.0, 507.4 |
|  | **Fixed-site exposures** |  |  |  |  |  |  | |  |
|  | PM_2.5_ (µg/m^3^) | 5.5 | 41/41 | 229.3 | -468.2, 926.7 | 39/39 | 202.2 | | -639.4, 1043.7 |
|  | Noise (dBA) | 5.2 |  | -14.5 | -437.1, 408.2 |  | -17.3 | | -503.6, 469.0 |
|  | NO_2_ (ppb) | 2.7 | 41/41 | 74.8 | -150.6, 300.3 | 39/39 | 84.7 | | -152.7, 322.2 |
|  | Noise (dBA) | 5.2 |  | -19.1 | -439.9, 401.7 |  | -26.6 | | -499.5, 446.4 |
|  | O_3_ (ppb) | 12.2 | 41/41 | 43.9 | -508.6, 596.4 | 39/39 | -3.42 | | -613.1, 606.2 |
|  | Noise (dBA) | 5.2 |  | -45.8 | -526.0, 434.4 |  | -30.6 | | -453.5, 392.3 |
|  | O_x_ (ppb) | 7.4 | 41/41 | 89.5 | -423.7, 602.6 | 39/39 | 58.9 | | -519.7, 637.6 |
|  | Noise (dBA) | 5.2 |  | -24.4 | -448.2, 602.6 |  | -35.5 | | -518.7, 447.8 |
|  |  |  |  |  |  |  |  | |  |
| **HF** | **Personal exposures** |  |  |  |  |  |  | |  |
|  | UFPs (particles/cm^3^) | 14,890 | 38/38 | -66.9 | -290.8, 156.9 | 36/36 | -70.5 | | -300.5, 159.4 |
|  | Noise (dBA) | 5.2 |  | -138.4 | -458.5, 181.7 |  | -122.3 | | -495.1, 250.5 |
|  | Black carbon (ng/m^3^) | 1,464 | 40/40 | -86.7 | -328.7, 155.4 | 38/38 | -48.9 | | -310.4, 212.6 |
|  | Noise (dBA) | 5.2 |  | -132.8 | -493.6, 227.9 |  | -163.7 | | -571.9, 244.5 |
|  | **Fixed-site exposures** |  |  |  |  |  |  | |  |
|  | PM_2.5_ (µg/m^3^) | 5.5 | 41/41 | 44.31 | -435.0, 523.6 | 39/39 | 154.6 | | -413.2, 722.5 |
|  | Noise (dBA) | 5.2 |  | -172.6 | -463.0, 117.8 |  | -143.8 | | -471.9, 184.3 |
|  | NO_2_ (ppb) | 2.7 | 41/41 | -34.1 | -188.7, 120.6 | 39/39 | -38.1 | | -199.2, 123.0 |
|  | Noise (dBA) | 5.2 |  | -183.1 | -471.7, 105.5 |  | -173.7 | | -494.6, 147.2 |
|  | O_3_ (ppb) | 12.2 | 41/41 | -34.1 | -411.6-343.4 | 39/39 | -19.0 | | -430.7, 392.8 |
|  | Noise (dBA) | 5.2 |  | -178.9 | -467.9, 110.1 |  | -167.7 | | -492.0, 156.6 |
|  | Ox (ppb) | 7.4 | 41/41 | -53.9 | -404.8, 296.9 | 39/39 | -46.6 | | -437.3, 344.2 |
|  | Noise (dBA) | 5.2 |  | -182.1 | -471.8, 107.7 |  | -173.0 | | -499.4, 153.3 |
|  |  |  |  |  |  |  |  | |  |
| **LF:HF** | **Personal exposures** |  |  |  |  |  |  | |  |
|  | UFPs (particles/cm^3^) | 14,890 | 38/38 | 0.270 | -0.546, 1.09 | 36/36 | 0.254 | | -0.560, 1.07 |
|  | Noise (dBA) | 5.2 |  | -0.083 | -1.25, 1.09 |  | 0.183 | -1.14, 1.50 | |
|  | Black carbon (ng/m^3^) | 1,464 | 40/40 | -0.225 | -1.23, 0.779 | 38/38 | -0.350 | -1.38, 0.68 | |
|  | Noise (dBA) | 5.2 |  | 0.358 | -1.14, 1.85 |  | 0.794 | -0.821, 2.41 | |
|  | **Fixed-site exposures** |  |  |  |  |  |  |  | |
|  | PM_2.5_ (µg/m^3^) | 5.5 | 41/41 | **-1.78** | **-3.66, 0.108** | 39/39 | **-1.83** | **-4.01-0.355** | |
|  | Noise (dBA) | 5.2 |  | 0.017 | -1.13, 1.16 |  | 0.081 | -1.18, 1.34 | |
|  | NO_2_ (ppb) | 2.7 | 41/41 | -0.260 | -0.894, 0.373 | 39/39 | -0.167 | -0.810-0.476 | |
|  | Noise (dBA) | 5.2 |  | 0.116 | -1.07, 1.30 |  | 0.298 | -0.982, 1.58 | |
|  | O_3_ (ppb) | 12.2 | 41/41 | -1.21 | -2.71, 0.298 | 39/39 | -1.05 | -2.65-0.282 | |
|  | Noise (dBA) | 5.2 |  | 0.077 | -1.07, 1.23 |  | 0.201 | -1.06, 1.46 | |
|  | Ox (ppb) | 7.4 | 41/41 | **-1.30** | **-2.68, 0.082** | 39/39 | -1.15 | -2.65-0.362 | |
|  | Noise (dBA) | 5.2 |  | 0.029 | -1.11, 1.17 |  | 0.143 | -1.12, 1.40 | |
| UFPs - ultrafine particles. IQR - interquartile range. Regression coefficients reflect the absolute change per IQR increase in exposure. ^*^n/N = visits/participants. Data reflect 1 study visit when HRV measures were taken (participants = visits). ^^^ adjusted for continuous temperature (degrees Celsius). ^^^^ adjusted for personal noise exposure (dBA), continuous temperature (degrees Celsius), alcohol intake (yes/no) and caffeine intake (yes/no) in the last 24 hours. | | | | | | | | | |

**Supplementary Table S7. Associations between repeated 30-minute average pollutant measurements and 5-minute HRV parameter measures, in single pollutant models.**

|  | | **Temperature-adjusted model^^^** | | | | **Fully-adjusted model^^^^** | | |
| --- | --- | --- | --- | --- | --- | --- | --- | --- |
| **Outcome** | **Exposure** | **IQR** | **n/N ^*^** | **Coefficient** | **95% Cl** | **n/N ^*^** | **Coefficient** | **95% CI** |
| **SDNN** | **Personal exposures** |  |  |  |  |  |  |  |
|  | UFPs (particles/cm^3^)^†^ | 5-fold | 418/42 | 0.69 | -2.36 – 3.73 | 397/40 | 0.60 | -2.48 – 3.68 |
|  | Black carbon (ng/m^3^)^†^ | 2.7-fold | 441/45 | **-3.63** | **-6.84 - -0.41** | 420/43 | **-3.66** | **-6.94 - -0.38** |
|  | Noise (dBA) | 8.4 | 353/42 | **-7.95** | **-12.3 - -3.59** | 332/40 | **-8.95** | **-13.5 - -4.42** |
|  | **Fixed-site exposures** |  |  |  |  |  |  |  |
|  | PM_2.5_ (µg/m^3^) | 7.9 | 455/46 | **-9.24** | **-15.5- -3.02** | 434/44 | **-8.69** | **-14.9- -2.47** |
|  | NO_2_ (ppb) | 4.0 | 455/46 | **-5.12** | **-9.01 - -1.23** | 434/44 | **-5.16** | **-9.14 - -1.19** |
|  | O_3_ (ppb) | 15.1 | 455/46 | **11.0** | **4.21 – 17.8** | 434/44 | **10.5** | **3.68 – 17.4** |
|  | O_x_ (ppb) | 8.7 | 455/46 | **9.05** | **2.60 – 15.5** | 434/44 | **6.90** | **1.06 – 12.7** |
|  |  |  |  |  |  |  |  |  |
| **RMSSD** | **Personal exposures** |  |  |  |  |  |  |  |
|  | UFPs (particles/cm^3^)^†^ | 5-fold | 418/42 | -0.14 | -1.58 – 1.31 | 397/40 | -0.09 | -1.56 – 1.37 |
|  | Black carbon (ng/m^3^)^†^ | 2.7-fold | 441/45 | **-4.43** | **-5.83 - -3.03** | 420/43 | **-4.47** | **-5.89 - -3.05** |
|  | Noise (dBA) | 8.4 | 353/42 | **-7.00** | **-8.84 - -5.14** | 332/40 | **-7.53** | **-9.45 - -5.61** |
|  | **Fixed-site exposures** |  |  |  |  |  |  |  |
|  | PM_2.5_ (µg/m^3^) | 7.9 | 455/46 | **-3.42** | **-6.63 - -0.213** | 434/44 | **-3.11** | **-6.35 - -0.12** |
|  | NO_2_ (ppb) | 4.0 | 455/46 | -0.73 | -2.70 – 1.24 | 434/44 | -0.74 | -2.76– 1.27 |
|  | O_3_ (ppb) | 15.1 | 455/46 | 2.22 | -1.25 – 5.68 | 434/44 | 1.93 | -1.60 – 5.45 |
|  | O_x_ (ppb) | 8.7 | 455/46 | 1.72 | -1.58 – 5.03 | 434/44 | 1.46 | -1.91 – 4.83 |
|  |  |  |  |  |  |  |  |  |
| **LF^**^** | **Personal exposures** |  |  |  |  |  |  |  |
|  | UFPs (particles/cm^3^)^†^ | 5-fold | 418/42 | 0.08 | -0.6 – 0.22 | 397/40 | 0.08 | -0.07 – 0.22 |
|  | Black carbon (ng/m^3^)^†^ | 2.7-fold | 445/45 | **-0.52** | **-0.68 - -0.35** | 424/43 | **-0.50** | **-0.66 - -0.34** |
|  | Noise (dBA) | 8.4 | 357/42 | **-0.69** | **-0.89 - -0.50** | 336/40 | **-0.71** | **-0.92 - -0.51** |
|  | **Fixed-site exposures** |  |  |  |  |  |  |  |
|  | PM_2.5_ (µg/m^3^) | 7.9 | 459/46 | -0.16 | -0.48 – 0.16 | 438/44 | -0.15 | -0.48 – 0.19 |
|  | NO_2_ (ppb) | 4.0 | 459/46 | -0.05 | -0.24 – 0.15 | 438/44 | -0.03 | -0.24 – 0.17 |
|  | O_3_ (ppb) | 15.1 | 459/46 | 0.19 | -0.11 – 0.50 | 438/44 | 0.21 | -0.12 – 0.55 |
|  | O_x_ (ppb) | 8.7 | 459/46 | 0.23 | -0.09 – 0.13 | 438/44 | 0.21 | -0.12 – 0.53 |
|  |  |  |  |  |  |  |  |  |
| **HF^**^** | **Personal exposures** |  |  |  |  |  |  |  |
|  | UFPs (particles/cm^3^)^†^ | 5-fold | 418/42 | 0.03 | -0.13 – 0.18 | 397/40 | 0.03 | -0.12 – 0.19 |
|  | Black carbon (ng/m^3^)^†^ | 2.7-fold | 445/45 | **-0.46** | **-0.62 - -0.31** | 424/43 | **-0.46** | **-0.62 - -0.30** |
|  | Noise (dBA) | 8.4 | 357/42 | **-0.79** | **-1.00 - -0.58** | 336/40 | **-0.81** | **-1.03 - -0.58** |
|  | **Fixed-site exposures** |  |  |  |  |  |  |  |
|  | PM_2.5_ (µg/m^3^) | 7.9 | 459/46 | **-0.32** | **-0.67 – 0.03** | 438/44 | **-0.31** | **-0.70 – 0.07** |
|  | NO_2_ (ppb) | 4.0 | 459/46 | 0.01 | -0.21 – 0.23 | 438/44 | 0.01 | -0.22 – 0.24 |
|  | O_3_ (ppb) | 15.1 | 459/46 | 0.05 | -0.29 – 0.38 | 43844 | 0.03 | -0.33 – 0.39 |
|  | O_x_ (ppb) | 8.7 | 459/46 | 0.05 | -0.27 – 0.38 | 438/44 | 0.03 | -0.32 – 0.38 |
|  |  |  |  |  |  |  |  |  |
| **LF:HF^**^** | **Personal exposures** |  |  |  |  |  |  |  |
|  | UFPs (particles/cm^3^)^†^ | 5-fold | 418/42 | 0.06 | -0.02 – 0.14 | 397/40 | 0.04 | -0.03 – 0.12 |
|  | Black carbon (ng/m^3^)^†^ | 2.7-fold | 445/45 | **0.11** | **0.03 – 0.19** | 424/43 | **0.11** | **0.03 – 0.19** |
|  | Noise (dBA) | 8.4 | 357/42 | **0.16** | **0.06 – 0.27** | 336/40 | **0.15** | **0.04 – 0.26** |
|  | **Fixed-site exposures** |  |  |  |  |  |  |  |
|  | PM_2.5_ (µg/m^3^) | 7.9 | 459/46 | **0.15** | **-0.01 – 0.32** | 438/44 | 0.14 | -0.03 – 0.31 |
|  | NO_2_ (ppb) | 4.0 | 459/46 | -0.001 | -0.10 – 0.10 | 438/44 | -0.01 | -0.11 – 0.09 |
|  | O_3_ (ppb) | 15.1 | 459/46 | 0.11 | -0.05 – 0.26 | 438/44 | 0.13 | -0.03 – 0.28 |
|  | O_x_ (ppb) | 8.7 | 459/46 | 0.585 | -0.206 – 1.38 | 438/44 | 0.65 | -0.138 – 1.43 |
|  |  |  |  |  |  |  |  |  |
| **Heart** | **Personal exposures** |  |  |  |  |  |  |  |
| **Rate** | UFPs (particles/cm^3^)^†^ | 5-fold | 418/42 | 0.06 | -1.85 – 1.98 | 397/40 | 0.10 | -1.82 – 2.04 |
|  | Black carbon (ng/m^3^)^†^ | 2.7-fold | 445/45 | **5.91** | **4.00 – 7.82** | 424/43 | **6.04** | **4.10 – 8.00** |
|  | Noise (dBA) | 8.4 | 357/42 | **10.8** | **8.34 – 13.3** | 336/40 | **11.3** | **8.7 – 13.8** |
|  | **Fixed-site exposures** |  |  |  |  |  |  |  |
|  | PM_2.5_ (µg/m^3^) | 7.9 | 459/46 | 3.83 | -1.16 – 8.82 | 438/44 | 3.42 | -1.72 – 8.56 |
|  | NO_2_ (ppb) | 4.0 | 459/46 | 0.292 | -2.42 – 3.01 | 438/44 | 0.25 | -2.52 – 3.02 |
|  | O_3_ (ppb) | 15.1 | 459/46 | -1.32 | -6.07 – 3.43 | 438/44 | -1.03 | -5.91 – 3.79 |
|  | O_x_ (ppb) | 8.7 | 459/46 | -1.01 | -5.55 – 3.53 | 438/44 | -0.80 | -5.45 – 3.85 |
| UFPs - ultrafine particles. IQR - interquartile range. ^†^UFP and BC exposures are log-base 5 and natural log transformed, respectively. ^*^n/N = visits/participants. ^**^LF, HF and LF:HF outcomes are natural log transformed. ^^^ adjusted for continuous temperature (degrees Celsius). ^^^^ adjusted for continuous temperature (degrees Celsius), alcohol intake in the last 24 hours (yes/no) and caffeine intake in the last 24 hours (yes/no). | | | | | | | | |

**Supplementary Table S8. Associations between repeated 30-minute average pollutant measurements and 5-minute HRV parameter measures, in two-pollutant models (air pollutant + noise).**

|  | | **Temperature-adjusted model^^^** | | | | **Fully-adjusted model^^^^** | | |
| --- | --- | --- | --- | --- | --- | --- | --- | --- |
| **Outcome** | **Exposure** | **IQR** | **n/N ^*^** | **Coefficient** | **95% Cl** | **n/N ^*^** | **Coefficient** | **95% CI** |
| **SDNN** | **Personal exposures** |  |  |  |  |  |  |  |
|  | UFPs (particles/cm^3^)^†^ | 5-fold | 330/38 | 0.69 | -2.66, 4.04 | 309/36 | 0.63 | -2.69,3.96 |
|  | Noise (dBA) | 8.4 |  | **-8.83** | **-13.31, -4.35** |  | **-9.97** | **-14.7, -5.29** |
|  | Black carbon (ng/m^3^)^†^ | 2.7-fold | 340/41 | -0.65 | -4.65, 3.34 | 319/39 | -0.34 | -4.39, 3.71 |
|  | Noise (dBA) | 8.4 |  | **-7.70** | **-12.8, -2.61** |  | **-8.99** | **-14.3, -3.65** |
|  | **Fixed-site exposures** |  |  |  |  |  |  |  |
|  | PM_2.5_ (µg/m^3^) | 7.9 | 353/42 | **-7.50** | **-13.8, -1.18** | 332/40 | **-6.86** | **-13.01, -0.72** |
|  | Noise (dBA) | 8.4 |  | **-7.93** | **-12.2, -3.61** |  | **-8.86** | **-13.4, -4.48** |
|  | NO_2_ (ppb) | 4.0 | 353/42 | **-3.23** | **-7.32, 0.87** | 332/40 | **-3.31** | **-7.42, 0.80** |
|  | Noise (dBA) | 8.4 |  | **-7.54** | **-11.9, -3.17** |  | -8.60 | -13.1, -4.07 |
|  | O_3_ (ppb) | 15.1 | 353/42 | **7.75** | **0.33, 15.2** | 332/40 | **6.18** | **-0.93, 13.3** |
|  | Noise (dBA) | 8.4 |  | **-7.26** | **-11.6, -2.90** |  | -8.30 | -12.8, -3.78 |
|  | O_x_ (ppb)^**^ | 8.7 | 353/42 | **5.89** | **-0.85, 12.6** | 332/40 | **4.29** | **-1.86, 10.4** |
|  | Noise (dBA) | 8.4 |  | **-7.51** | **-11.9, -3.16** |  | **-8.64** | **-13.2, -4.12** |
|  |  |  |  |  |  |  |  |  |
| **RMSSD** | **Personal exposures** |  |  |  |  |  |  |  |
|  | UFPs (particles/cm^3^)^†^ | 5-fold | 330/38 | 0.67 | -0.81, 2.14 | 309/36 | 0.84 | -0.62, 2.30 |
|  | Noise (dBA) | 8.4 |  | **-7.77** | **-9.68, -5.85** |  | **-8.53** | **-10.5, -6.54** |
|  | Black carbon (ng/m^3^)^†^ | 2.7-fold | 340/41 | **-2.52** | **-4.15, -0.89** | 319/39 | **-2.39** | **-4.06, -0.73** |
|  | Noise (dBA) | 8.4 |  | **-5.64** | **-7.74, -3.53** |  | **-6.24** | **-8.45, -4.02** |
|  | **Fixed-site exposures** |  |  |  |  |  |  |  |
|  | PM_2.5_ (µg/m^3^) | 7.9 | 353/42 | **-2.92** | **-5.98, 0.15** | 332/40 | **-2.70** | **-5.70, 0.30** |
|  | Noise (dBA) | 8.4 |  | **-7.00** | **-8.84, -5.15** |  | **-7.57** | **-9.48, -5.66** |
|  | NO_2_ (ppb) | 4.0 | 353/42 | 0.024 | -1.87, 1.92 | 332/40 | -0.006 | -1.91, 1.90 |
|  | Noise (dBA) | 8.4 |  | **-6.99** | **-8.85, -5.14** |  | **-7.53** | **-9.46, -5.61** |
|  | O_3_ (ppb) | 15.1 | 353/42 | 0.61 | -3.00, 4.21 | 332/40 | -0.12 | -3.72, 3.48 |
|  | Noise (dBA) | 8.4 |  | **-7.06** | **-8.92, -5.20** |  | **-7.50** | **-9.43, -5.56** |
|  | O_x_ (ppb)^**^ | 8.7 | 353/42 | 0.20 | -3.20, 3.61 | 332/40 | -0.49 | -3.90, 2.92 |
|  | Noise | 8.4 |  | **-7.04** | **-8.90, -5.19** |  | **-7.52** | **-9.45, -5.60** |
|  |  |  |  |  |  |  |  |  |
| **LF**^**^ | **Personal exposures** |  |  |  |  |  |  |  |
|  | UFPs (particles/cm^3^)^†^ | 5-fold | 330/38 | **0.15** | **0.007, 0.30** | 309/36 | **0.16** | **0.009, 0.30** |
|  | Noise (dBA) | 8.4 |  | **-0.75** | **-0.94, -0.55** |  | **-0.77** | **-0.97, -0.57** |
|  | Black carbon (ng/m^3^)^†^ | 2.7-fold | 344/41 | -0.14 | -0.31, 0.03 | 323/39 | **-0.11** | **-0.29, 0.06** |
|  | Noise (dBA) | 8.4 |  | **-0.63** | **-0.85, -0.42** |  | **-0.67** | **-0.90, -0.43** |
|  | **Fixed-site exposures** |  |  |  |  |  |  |  |
|  | PM_2.5_ (µg/m^3^) | 7.9 | 357/42 | -0.07 | -0.39, 0.24 | 336/40 | -0.06 | -0.38, 0.27 |
|  | Noise (dBA) | 8.4 |  | **-0.67** | **-0.87, -0.48** |  | **-0.69** | **-0.89**, **-0.49** |
|  | NO_2_ (ppb) | 4.0 | 357/42 | -0.004 | -0.21, 0.20 | 336/40 | -0.008 | -0.22, 0.21 |
|  | Noise (dBA) | 8.4 |  | **-0.68** | **-0.88, -0.49** |  | **-0.71** | **-0.91**, **-0.50** |
|  | O_3_ (ppb) | 15.1 | 357/42 | 0.03 | -0.27, 0.33 | 336/40 | 0.004 | -0.31, 0.32 |
|  | Noise (dBA) | 8.4 |  | **-0.67** | **-0.87, -0.48** |  | **-0.69** | **-0.90**, **-0.49** |
|  | O_x_ (ppb)^**^ | 8.7 | 357/42 | -0.08 | -0.41, 0.25 | 336/40 | -0.13 | -0.47, 0.21 |
|  | Noise (dBA) | 8.4 |  | **-0.78** | **-0.99, -0.57** |  | **-0.80** | **-1.02**, **-0.58** |
|  |  |  |  |  |  |  |  |  |
| **HF**^**^ | **Personal exposures** |  |  |  |  |  |  |  |
|  | UFPs (particles/cm^3^)^†^ | 5-fold | 330/38 | 0.09 | -0.08, 0.25 | 309/36 | 0.11 | -0.06, 0.27 |
|  | Noise (dBA) | 8.4 |  | **-0.86** | **-1.08, -0.64** |  | **-0.89** | **-1.12, -0.66** |
|  | Black carbon (ng/m^3^)^†^ | 2.7-fold | 344/41 | **-0.20** | **-0.38, -0.02** | 323/39 | **-0.19** | **-0.37, 0.002** |
|  | Noise (dBA) | 8.4 |  | **-0.71** | **-0.95, -0.47** |  | **-0.74** | **-0.99, -0.49** |
|  | **Fixed-site exposures** |  |  |  |  |  |  |  |
|  | PM_2.5_ (µg/m^3^) | 7.9 | 357/42 | -0.23 | -0.57, 0.12 | 336/40 | -0.21 | -0.56, 0.13 |
|  | Noise (dBA) | 8.4 |  | **-0.77** | **-0.99, -0.56** |  | **-0.79** | **-1.02, -0.57** |
|  | NO_2_ (ppb) | 4.0 | 357/42 | 0.04 | -0.19, 0.27 | 336/40 | 0.03 | -0.20, 0.27 |
|  | Noise (dBA) | 8.4 |  | **-0.78** | **-1.00, -0.57** |  | **-0.81** | **-1.03, -0.59** |
|  | O_3_ (ppb) | 15.1 | 357/42 | -0.09 | -0.43, 0.25 | 336/40 | -0.14 | -0.49, 0.21 |
|  | Noise (dBA) | 8.4 |  | **-0.78** | **-0.99, -0.57** |  | **-0.80** | **-1.03, -0.58** |
|  | O_x_ (ppb)^**^ | 8.7 | 357/42 | -0.08 | -0.41, 0.25 | 336/40 | -0.13 | -0.47, 0.21 |
|  | Noise (dBA) | 8.4 |  | -0.78 | -0.99 - -0.57 |  | -0.80 | -1.02, -0.58 |
|  |  |  |  |  |  |  |  |  |
| **LF:HF**^**^ | **Personal exposures** |  |  |  |  |  |  |  |
|  | UFPs (particles/cm^3^)^†^ | 5-fold | 330/38 | 0.07 | -0.01, 0.14 | 309/36 | 0.05 | -0.03, 0.13 |
|  | Noise (dBA) | 8.4 |  | **0.17** | **0.06, 0.28** |  | **0.17** | **0.06, 0.28** |
|  | Black carbon (ng/m^3^)^†^ | 2.7-fold | 344/41 | 0.06 | -0.03, 0.16 | 323/39 | 0.06 | -0.03, 0.16 |
|  | Noise (dBA) | 8.4 |  | **0.13** | **0.01, 0.26** |  | **0.13** | **0.01, 0.25** |
|  | **Fixed-site exposures** |  |  |  |  |  |  |  |
|  | PM_2.5_ (µg/m^3^) | 7.9 | 357/42 | **0.15** | **-0.01, 0.31** | 336/40 | **0.13** | **-0.03, 0.29** |
|  | Noise (dBA) | 8.4 |  | **0.16** | **0.05, 0.26** |  | **0.15** | **0.04, 0.26** |
|  | NO_2_ (ppb) | 4.0 | 357/42 | -0.02 | -0.12, 0.08 | 336/40 | -0.02 | -0.13, 0.08 |
|  | Noise (dBA) | 8.4 |  | **0.16** | **0.05, 0.26** |  | **0.15** | **0.05, 0.26** |
|  | O_3_ (ppb) | 15.1 | 357/42 | 0.11 | -0.05, 0.27 | 336/40 | 0.13 | -0.03, 0.29 |
|  | Noise (dBA) | 8.4 |  | **0.16** | **0.06, 0.27** |  | **0.15** | **0.05, 0.26** |
|  | O_x_ (ppb)^**^ | 8.7 | 357/42 | 0.11 | -0.04, 0.27 | 336/40 | 0.13 | -0.03, 0.29 |
|  | Noise (dBA) | 8.4 |  | **0.16** | **0.06, 0.27** |  | **0.16** | **0.05, 0.26** |
|  |  |  |  |  |  |  |  |  |
| **Heart** | **Personal exposures** |  |  |  |  |  |  |  |
| **Rate** | UFPs (particles/cm^3^)^†^ | 5-fold | 330/38 | -1.97 | -3.08, 0.68 | 309/36 | -1.24 | -3.11**,** 0.62 |
|  | Noise (dBA) | 8.4 |  | **11.8** | **9.44, 14.2** |  | **12.3** | **9.8, 14.8** |
|  | Black carbon (ng/m^3^)^†^ | 2.7-fold | 344/41 | 1.13 | -1.03, 3.28 | 323/39 | 1.05 | -1.15, 3.26 |
|  | Noise (dBA) | 8.4 |  | **10.5** | **7.80, 13.3** |  | **11.0** | **8.17, 13.9** |
|  | **Fixed-site exposures** |  |  |  |  |  |  |  |
|  | PM_2.5_ (µg/m^3^) | 7.9 | 357/42 | 2.02 | -2.59, 6.62 | 336/40 | 1.75 | -2.95, 6.45 |
|  | Noise (dBA) | 8.4 |  | **10.5** | **8.09, 13.0** |  | **10.9** | **8.39, 13.5** |
|  | NO_2_ (ppb) | 4.0 | 357/42 | -0.67 | -3.18, 1.83 | 336/40 | -0.58 | -3.12, 1.67 |
|  | Noise (dBA) | 8.4 |  | **10.7** | **8.24, 13.2** |  | **11.0** | **8.49, 13.6** |
|  | O_3_ (ppb) | 15.1 | 357/42 | 2.17 | -1.91, 6.25 | 336/40 | 2.25 | -1.82, 6.30 |
|  | Noise (dBA) | 8.4 |  | **10.7** | **8.26, 13.2** |  | **11.1** | **8.58, 13.7** |
|  | O_x_ (ppb)^**^ | 8.7 | 357/42 | 2.14 | -1.97, 6.24 | 336/40 | 2.28 | -1.74, 6.30 |
|  | Noise (dBA) | 8.4 |  | **10.7** | **8.21, 13.1** |  | **11.1** | **8.57, 13.7** |
| UFPs - ultrafine particles. IQR - interquartile range. ^†^UFP and BC exposures are log-base 5 and natural log transformed, respectively. ^*^n/N = visits/participants. ^**^LF, HF and LF:HF outcomes are natural log transformed. ^^^ adjusted for continuous temperature (degrees Celsius). ^^^^ adjusted for continuous temperature (degrees Celsius), alcohol intake (yes/no) and caffeine intake (yes/no) in the last 24 hours. | | | | | | | | |

**Supplementary Table S9. Associations between repeated 30-minute average pollutant measurements and 5-minute HRV parameter measures, in models stratified by sex, personal noise exposure (<68.1 vs ≥68.1 dBA) and Ox (<23.4 ppb vs. ≥ 23.4 ppb).**

|  | |  | **Females** | | | **Males** | | | |
| --- | --- | --- | --- | --- | --- | --- | --- | --- | --- |
| **Outcome** | **Exposure** | **IQR** | **N** | **Coefficient** | **95% Cl** | **N** | **Coefficient** | **95% CI** | **p-interaction** |
| **Heart Rate** | Black carbon (ng/m^3^)^†^ | 2.7-fold | 30 | **7.33** | **4.95, 9.71** | 13 | **3.45** | **0.17, 6.74** | 0.06 |
|  | PM_2.5_ (µg/m3) | 7.9 | 31 | **7.55** | **1.48, 13.6** | 13 | -0.01 | -6.66, 6.46 | 0.09 |
| **SDNN** | Black carbon (ng/m^3^)^†^ | 2.7-fold | 30 | **-6.72** | **-10.8, -2.68** | 13 | 2.12 | -3.46, 7.70 | 0.01 |
| **RMSSD** | Black carbon (ng/m^3^)^†^ | 2.7-fold | 30 | **-6.47** | **-8.21, -4.74** | 13 | -0.79 | -3.16, 1.58 | 0.00 |
|  | Noise (dBA) | 8.4 | 28 | **-8.91** | **-11.3, -6.48** | 12 | **-5.25** | **-8.37, -2.13** | 0.07 |
| **HF ^**^** | Black carbon (ng/m^3^)^†^ | 2.7-fold | 30 | -0.66 | -0.66, -0.85 | 13 | -0.1 | -0.10, -0.36 | 0.00 |
|  | PM_2.5_ (µg/m^3^) | 7.9 | 31 | -0.64 | -0.64, -1.11 | 13 | 0.06 | 0.06, -0.45 | 0.05 |
| **LF:HF ^**^** | Black carbon (ng/m^3^)^†^ | 2.7-fold | 30 | 0.21 | 0.21, 0.12 | 13 | -0.08 | -0.08, -0.22 | 0.00 |
|  | PM_2.5_ (µg/m^3^) | 7.9 | 31 | 0.31 | 0.31, 0.09 | 13 | -0.05 | -0.05, -0.29 | 0.03 |
|  |  |  | **Noise <68.1 dBA** | | | **Noise ≥68.1 dBA** | | | |
| **Heart Rate** | PM_2.5_ (µg/m^3^) | 7.9 | 38 | 1.12 | -4.40, 6.64 | 44 | **5.44** | **0.14, 10.7** | 0.08 |
| **SDNN** | PM_2.5_ (µg/m^3^) | 7.9 | 38 | -3.11 | -11.0, 4.76 | 44 | **-13.2** | **-20.5, -5.93** | 0.02 |
| **RMSSD** | UFPs (particles/cm^3^)^†^ | 5-fold | 36 | 1.48 | -0.54, 3.49 | 40 | -0.85 | -2.51, 0.81 | 0.04 |
| **LF ^**^** | UFPs (particles/cm^3^)^†^ | 5-fold | 36 | 0.21 | 0.02, 0.40 | 40 | 0.02 | -0.14, 0.18 | 0.09 |
|  | PM_2.5_ (µg/m^3^) | 7.9 | 38 | 0.04 | -0.33, 0.42 | 44 | -0.30 | -0.66, 0.05 | 0.06 |
| **HF ^**^** | PM_2.5_ (µg/m^3^) | 7.9 | 38 | 0.01 | -0.04, 0.43 | 44 | -0.59 | -0.98, -0.19 | 0.00 |
| **LF:HF ^**^** | PM_2.5_ (µg/m^3^) | 7.9 | 38 | 0 | -0.20, 0.20 | 44 | 0.25 | 0.06, 0.44 | 0.01 |
|  | O_3_ (ppb) | 15.1 | 38 | 0 | -0.20, 0.20 | 44 | 0.18 | 0.01, 0.34 | 0.06 |
|  | O_x_ (ppb) | 8.7 | 38 | 0.01 | -0.18, 0.21 | 44 | 0.18 | 0.02, 0.35 | 0.05 |
|  |  |  | **Ox<23.4 ppb** | | | **Ox ≥ 23.4 ppb** | | | |
| **Heart Rate** | UFPs (particles/cm^3^)^†^ | 5-fold | 35 | -2.09 | -4.65, 0.46 | 32 | **1.84** | **-0.70, 4.38** | 0.02 |
|  | PM_2.5_ (µg/m^3^) | 7.9 | 38 | -0.58 | -7.18, 6.02 | 34 | **6.17** | **0.56, 11.8** | 0.03 |
| **LF ^**^** | UFPs (particles/cm^3^)^†^ | 5-fold | 35 | 0.22 | 0.03, 0.41 | 32 | -0.02 | -0.21, 0.17 | 0.06 |
|  | PM_2.5_ (µg/m^3^) | 7.9 | 38 | 0.12 | -0.33, 0.57 | 34 | -0.34 | -0.34, -0.71 | 0.05 |
| **HF ^**^** | UFPs (particles/cm^3^)^†^ | 5-fold | 35 | 0.17 | -0.04, 0.38 | 32 | -0.08 | -0.08, -0.29 | 0.09 |
| **LF:HF^**^** | PM_2.5_ (µg/m^3^) | 7.9 | 38 | 0.35 | 0.12, 0.58 | 34 | 0.04 | 0.04, -0.14 | 0.01 |
| UFPs - ultrafine particles. IQR - interquartile range. Models adjusted for temperature, 24-hour caffeine intake (yes/no) and 24-hour alcohol intake (yes/no). Associations are presented only for models where an interaction was detected (initial threshold value of p-interaction<0.10). We then examined stratified estimates and confidence intervals, for evidence of meaningful differences across strata. †UFP and BC exposures are log-base 5 and natural log transformed, respectively. ^**^LF, HF and LF:HF outcomes are natural log transformed. | | | | | | | | | |

**Summary of results for fixed-site regional pollutants:**

*Associations between pollution and acute (baseline to follow-up) within-person changes in cardiovascular outcomes*

Outdoor regional PM_2.5_, O_3_, and O_x_ were each associated with significant decreases in RHI and with small increases in diastolic blood pressure (Supplementary Figure 1). None of the pollutants were associated with changes in heart rate on the same day for the daily average exposures. Fixed-site regional pollutant concentrations were not associated with marked changes in HRV, however, these regional pollutants (PM_2.5_, O_3_ and O_x_) were associated with decreases in the LF:HF ratio. Associations between endothelial function and PM_2.5_, O_3_ and O_x_ were negligibly impacted by adjustment for noise exposure (slightly strengthened for PM_2.5_ and slightly attenuated for O_3_ and O_x_). The increase in diastolic BP with PM_2.5_ exposure detected in single pollutant models also persisted with adjustment for noise. Inverse associations between the LF:HF and PM_2.5_ and O_x_ were unchanged with noise adjustment (Supplementary Figure 2).

*Associations between pollution (30-minute average exposure) and subacute cardiovascular outcomes*

All regional fixed-site pollutants (PM_2.5_, NO_2_, O_3_ and O_x_) were associated with changes in SDNN; PM_2.5_ and NO_2_ were associated with significant decreases in SDNN, while O_3_ and O_x_ were associated with significant increases in this parameter. Lastly, regional fixed-site PM_2.5_ was associated with a reduction in RMSSD and HF. For the associations of 30-minute exposures to PM_2.5_ and NO_2_ with SDNN decreases, and those of O_3_ and O_x_ with SDNN increases, adjustment for noise attenuated the effect estimates for these exposures in two-pollutant models compared to single pollutant models. The marked decreases in concurrent SDNN and RMSSD in association with PM_2.5_ (% change of- 6.86 per 7.87 µg/m^3^, 95% CI -13.01 to -0.72 and -2.70 per 7.87 µg/m^3^, 95% CI-5.70 to +0.30, respectively) and in SDNN in association with NO_2_ (% change of -3.31 per 4.03 ppb, 95% CI -7.42 to +0.80) were still present with noise adjustment.


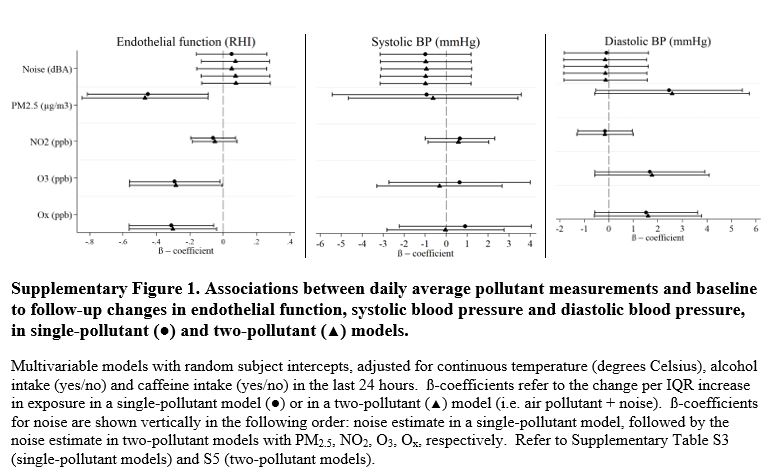


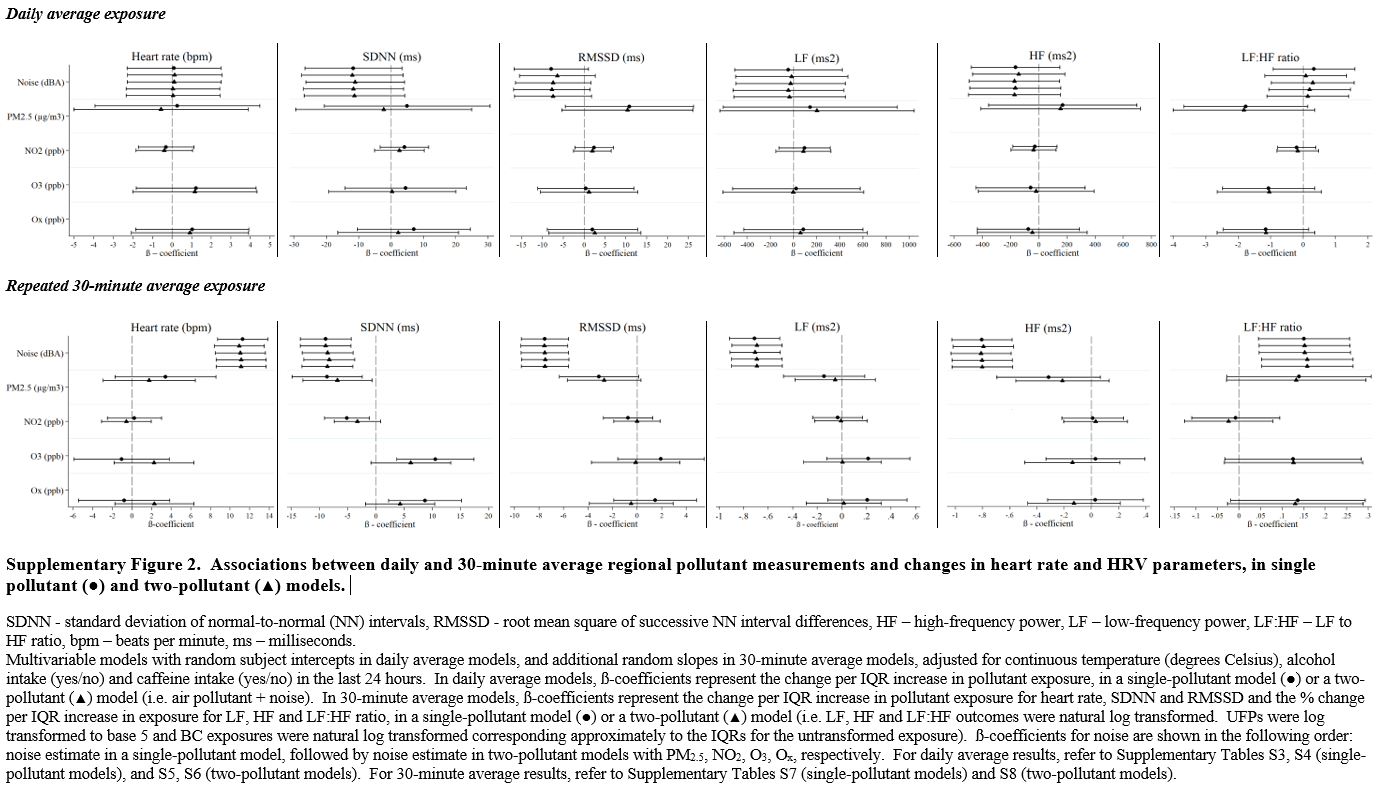

Supplement: Supplementary file 1 — Supplementary Information. [file 41598_2020_73412_MOESM1_ESM.docx]
